# Academic history, domains and distribution of the hot-cold system in Mexico

**DOI:** 10.1186/s13002-023-00624-1

**Published:** 2023-11-02

**Authors:** Karina Yaredi García-Hernández, Luis Alberto Vargas-Guadarrama, Heike Vibrans

**Affiliations:** 1 Posgrado en Botánica, Colegio de Postgraduados, 56264 Montecillo, Texcoco, Mexico State Mexico; 2 Instituto de Investigaciones Antropológicas, Universidad Nacional Autónoma de México, Circuito Exterior, Ciudad Universitaria, Coyoacán, 04510 Mexico City, Mexico

**Keywords:** Humoral system, Hot–cold classification, Academic history, Ethnobotany, Traditional medicine, Medicinal plants, Traditional food, Knowledge domains, Mesoamerica

## Abstract

**Background:**

The hot–cold classification system for things and concepts is widely used by many human groups in Mexico. We conducted a comprehensive review to understand the history, themes, and distribution of this system.

**Methods:**

We analyzed publications based on field work in Mexico, considering publication date, research approach, study depth, and conceptual domains. We identified the ethnic groups that use the system and the places where they live. A map illustrates the geographic and cultural distribution of the system.

**Results:**

The hot–cold system has been documented in 101 academic publications spanning almost a century, particularly for traditional medicine and food. Initially dominated by anthropological studies, ethnobotanists have increasingly contributed to the research. The hot–cold system is utilized by at least 56 indigenous ethnic groups (81% of the total) and mestizos (whose primary or sole language is Spanish) across most of Mexico.

**Discussion:**

Anthropologists laid the foundation for understanding the hot–cold system, on which current ethnobotany builds. However, there are still knowledge gaps, for example on some domains (human beings, landscape) and on patterns by regions or linguistic families. The geographic and cultural distribution presented here is approximate, as group ethnicity is imprecise.

**Conclusions:**

The hot–cold system is widely applied in Mexico, although some variations exist. Further exploration of understudied domains, and variations between ethnic groups and regions, would contribute to a comprehensive explanation of this interconnected worldview.

**Supplementary Information:**

The online version contains supplementary material available at 10.1186/s13002-023-00624-1.

## Background

Dualism is a prevailing concept in many cultures around the world. It maintains that the world is structured by two opposing forces. Chinese cosmology embodies this through the concept of yin and yang [1]. ‘Yanantin’ is central to Andean Quechua philosophy and refers to harmonious relationships between complementary opposites like dark/light, exterior/interior, and notably, masculine/feminine [2]. The Mesoamerican tradition divides the world into complementary opposites: light/dark, dry/wet, high/low [3]. The Mediterranean humoral doctrine, that included the double opposites hot/cold and wet/dry, is also related [4].

A prominent contemporary example of dualism is the traditional classification of health issues, foods, and remedies into “hot” and “cold” categories. This division is observed globally, including in Southeast Asia [5], India [6], the Peruvian Andes [7, 8], the Greater Antilles [9, 10], and southeastern Brazil [11].

In most of Mexico, people also empirically classify objects, phenomena, and concepts into “hot” and “cold.” These categories are considered intrinsic properties or part of the nature of things, and are not always associated with temperature [12–14]. The classification system is particularly evident in traditional foodways, concepts of diseases and their treatment; it is applied to many, but not all, health problems, remedies, and foods (see, for example, [15, 16]). Treatments follow the principle of opposites: diseases considered “hot” are treated with “cold” remedies, and vice versa [14, 17, 18]. However, this general rule may be modified; the hot–cold system is highly versatile and complex [16, 19, 20]. In addition, the system is applied to other domains, such as colors, times of the year, human life stages, elements of the environment, and emotions [19].

The origin of the system in Mesoamerica has been somewhat controversial. George Foster [21, 22] suggested an origin in the European humoral theory. In contrast, Alfredo López Austin [23] proposed the ideological principle of universe duality of the pre-Hispanic Mesoamerican peoples as source. Today, most researchers consider the system a result of syncretism between Mediterranean European concepts from the sixteenth century and the native Mesoamerican dual cosmology [24–26].

Anthropologists have documented the hot–cold system extensively for many years in different regions and human groups in Mexico. Notable examples include the Purepechas [27–30], the Zapotecs [13, 31–33], and the Nahuas [34, 35]. Ethnobotanists have also been interested in this topic, although to a lesser extent. Some studies [15, 36, 37] have found classification rules in medicinal plants, often linked to perceptible characteristics such as smell, taste, and habitat.

A recent literature review on the topic [19] showed that the hot–cold system is used by mestizo peoples and 29 ethno-linguistic groups mainly in the Mesoamerican area of Mexico. The information for this review was gathered during the first year of the pandemic (2020), so unfortunately, information from the *Library of Traditional Mexican Medicine*, a 12-volume encyclopedia published from 1990 to 1994 on traditional medicine of the indigenous peoples of Mexico, was inaccessible. Its online version (http://www.medicinatradicionalmexicana.unam.mx) could not be consulted either, as the website was inactive from April 2019 to January 2021 (information provided by site maintenance personnel of the National University of Mexico via email on March 29, 2023).

This study aims to (1) provide a historical overview of the documentation of the hot–cold system in Mexico; (2) identify the most frequently studied domains; and (3) update and understand the geographical and cultural scope of the hot–cold system in Mexico by integrating information from the *Digital Library of Mexican Traditional Medicine.* We propose guidelines and considerations for the ethnobotanical study of the hot–cold system.

## Methods

### Historical and subject analysis of the documentation of the hot–cold system in Mexico

Our data derive mostly from a bibliographic database assembled for previous work [16, 19, 20]. We selected publications that were based, at least partially, on fieldwork in Mexico. It was supplemented by searches in Google Scholar, SCOPUS, and Web of Science using keywords such as “humoral classification,” “hot–cold system,” “hot–cold classification,” “Mesoamerica,” and “Mexico” in both Spanish and English during 2022/2023.

The data extraction focused on the year of the original publication, the research approach (anthropological, ethnobotanical, or other), study depth (details or only mention), and the covered conceptual domains. For a publication to be considered ethnobotanical, plants had to play a prominent role in the research and include their taxonomic identification. A study covered a domain if it included information on the system of at least one of the following aspects:

- *Traditional medicine*: classification of diseases, remedies, transient states of the body (due to sun exposure, rain, ambient temperature, anger), life stages (childhood, adulthood), reproductive stages (menstruation, pregnancy), and others; and the explanation of knowledge, beliefs, and practices in the etiology, diagnosis, treatment, and prevention of diseases.
- *Food*: classification of foods of different origins (animal, vegetable, or mineral), criteria for classification, influence of cooking methods or preparation, and others; also, the description of diet during pregnancy, menstruation, and postpartum period.
- *Landscape*: classification of the landscape at the regional or community level.
- *People*: classification of individuals based on gender, skin color, personality, or other permanent attributes.
- *Others*: domains such as emotions (anger, sadness, sexual desire), elements of the environment (water, sun, rain, hail, soil), seasons of the year, celestial bodies, everyday objects (footwear, utensils), minerals, metals, soil types, and aspects related to agriculture.

The bibliographic references and the extracted data from all used publications can be found in sections A and B of Additional file 1.

### The geographic and cultural distribution of the hot–cold system in Mexico

To identify the ethnic groups using the hot–cold system and their geographic locations, we reviewed academic publications, and also monographs on indigenous communities in Mexico published by government sources in the *Atlas of Indigenous Peoples of Mexico* [38] and the collection *Indigenous Peoples of Contemporary Mexico* published from 2000 to 2010 by the former *National Commission for the Development of Indigenous Peoples* (CDI). We also consulted the online encyclopedia *Digital Library of Traditional Mexican Medicine*, specifically the sections on *Indigenous Medicinal Flora of Mexico* [39] and *Traditional Medicine of Indigenous Peoples of Mexico* [40]. These sources will be collectively referred to as monographs.

Traditional medicine was the only domain covered in the reviewed monographs. The *Atlas of Indigenous Peoples of Mexico* [38] and the collection *Indigenous Peoples of Contemporary Mexico* only reported on the system's use. The information in the *Indigenous Medicinal Flora of Mexico* [39] and *Traditional Medicine of Indigenous Peoples of Mexico* [40] was more detailed and included the classification of various plants and diseases (although not all listed), as well as related beliefs and practices.

The ethnic classification of the human groups was based on language, following the standard of the *Catalog of National Indigenous Languages* [41], which recognizes 68 linguistic groupings (or languages) belonging to 11 indigenous language families. When researchers emphasized that the population in their study area was predominantly mestizo or Spanish monolingual, they were classified as “mestizos”.

If studies did not report the languages spoken by the informants, the researchers' perception of ethnic or linguistic affiliation was taken into account. In some cases, self-identification was used as the basic criterion (admitted by Mexican law [42]). For example, the Cochimi people self-identify as indigenous despite their language being extinct and are recognized by the government [38]; the municipality of Coatetelco in the state of Morelos was declared “indigenous” based on its Nahua background [43], even though less than 1% of the population speak the language [44]. Nahuatl, Zapotec, and Mixtec speakers exhibit considerable internal cultural and linguistic diversity and were further subdivided based on the observations of Valiñas Coalla [45].

The fieldwork localities or the distribution areas of the indigenous communities were identified. If specific localities were not mentioned, we used municipal seats. We updated the nomenclature for communities, municipalities and states with the government database *Historical Archive of Geo-statistical Localities* [46]. The study sites were mapped with ArcGIS 10.8® software and differentiated by ethnic group. If several localities inhabited by the same ethnic group were in close proximity, within a 25 km radius, only one representative point was used.

Sections B-F of the supplementary material (Additional file 1) contain the relevant information from all consulted sources. Fifteen sources did not provide the geographic location of the studied human groups, so they were omitted from the map, except in some cases indicated in sections E and F. However, they were included in the analysis of cultural distribution. Three academic publications indicated in section B were excluded from both analyses because they did not specify the studied human groups or their geographic location.

The names of the ethnic groups and their languages can be found in section G of Additional file 1. As most of the names were exonyms, we also included endonyms. Section H provides details on the subdivision of the Nahua, Mixtec, and Zapotec groups. The information is organized first by language families and then by linguistic groupings, following the order of the *Catalog of National Indigenous Languages* [41].

To estimate the documentation level for different ethnic groups, we counted the sources used in this study. Each publication (academic or monographic) or individual web page of online monographs was considered one unit (for more details, see sections G and H of Additional file 1).

## Results

### Academic literature on the hot–cold system in Mexico: history

Of 101 academic publications documenting the use of the hot–cold system in Mexico, 63 were papers in scientific journals, 22 books, 11 book chapters and 3 conferences or symposia communications. Most had an anthropological focus (62%), often ethnographic, and another third were ethnobotanical studies (34%). A few studies were social, ethnozoological or ethnoecological.

The first reference (1930) to the hot–cold system was found in “Tepoztlan: A Mexican Village: A Study of Folk Life”, a classic work of the renowned anthropologist Robert Redfield [47]. It says on postpartum women “The patient cannot take food that is very hot or very cold”.

**Fig. 1:**
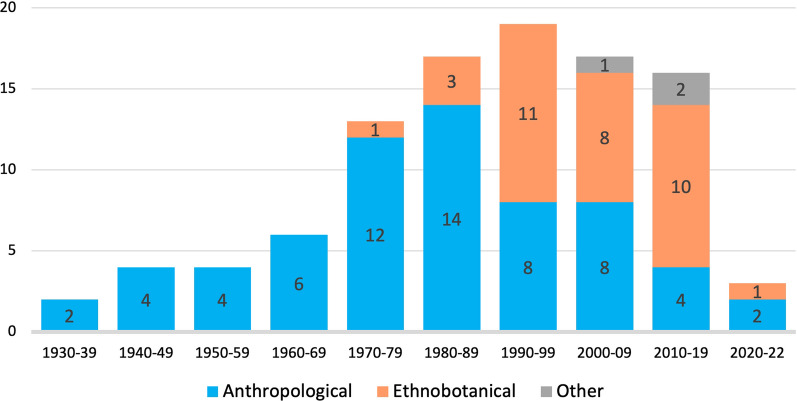
Number of publications documenting the hot–cold system by decade and study approach

Anthropological publications on the subject increased slightly from the 1940s to 1960s, and surged in the 1970s; this decade also saw the first ethnobotanical study. Between the 1980s and today the number of publications were relatively similar, but the proportion of ethnobotanical work increased steadily (see Fig. 1).

The nationality of the authors changed over time. Until the late 1980s, mainly American anthropologists worked on the topic; George M. Foster stood out. He briefly addressed the subject in his first two publications [48, 49], and described the system in detail almost two decades later, in a chapter in “Tzintzuntzan: Mexican Peasants in a Changing World” [30]. Foster systematized and integrated his in-depth research in Mexico and other regions in the book “Hippocrates' Latin American Legacy: Humoral Medicine in the New World” [50]. In this work, he acknowledged that he had underestimated the subject in his early studies.

American anthropologist Ellen Messer published the first relevant ethnobotanical study in 1978 [33]. During the 1990s, and parallel to the growth of ethnobotany as a discipline in Mexico [51], researchers from various disciplines and nationalities developed an interest in the topic. The German pharmaceutical biologist Michael Heinrich [52, 53] was among the pioneers, paving the way for European researchers to explore the hot–cold classification in medicinal and food plants through a mainly ethnopharmacological lens [15, 37, 54–56]. In this millennium (2000–2019), Mexican and foreign researchers published in approximately equal proportions.

### Academic literature on the hot–cold system in Mexico: main themes

Most publications (79%) either focused directly on the hot–cold system or included relevant details, while the remaining publications only mentioned its use in their study areas. Approximately half (50) explored multiple domains. Traditional medicine was the most frequently addressed topic (81%), followed by food (53%), landscape (17%), and people (14%). Additionally, 17% of the publications covered at least one “other” domain (emotions, color, etc.; Table 1).

**Table 1:** Number of academic publications by research approach and domain covered

Domain	Research approach		
	Anthropological	Ethnobotanical	Other
Traditional medicine	52	28	2
Food	42	10	1
People	14	0	0
Landscape	8	7	2
Other	16	1	0

“Other” research approaches include social science different from anthropology, ethnozoological or ethnoecological works. “Other” domains include various, such as soil types, seasons, emotions or colors

Ethnobotanical studies have paid less attention to the food (Table 1). The classification of people has been addressed exclusively by anthropologists, while anthropology and ethnobotany had a similar number of publications on landscape. Other domains have been documented primarily through anthropological studies ([19] for a review), in particular detail by Madsen ([35, 57].

Publications on traditional medicine primarily discussed the hot–cold classification of diseases and remedies, and how they relate through the principle of opposites. Some sources briefly reported facts [58–60], while others provided detailed descriptions and extensive lists of diseases and remedies with their respective hot–cold properties [18, 34, 36]. The latter type of publication offered insights into the classification mechanics as well as the associated knowledge, beliefs, and practices. A few ethnobotanical studies [16, 61–63] included lists of medicinal plants with their hot–cold properties and taxonomic identification.

Descriptions of traditional medicine often include information on food, as it can cause diseases or help to heal [13, 64, 65]. However, some studies focus on food and related aspects. Two topics that have attracted anthropologists' attention for some time are the intracultural variation in food classification [50, 66, 67] and the factors involved in the cognitive process of classification, such as classificatory criteria and the learning process [32, 68, 69]. Apparently, there is only one ethnobotanical study, on the classification of edible plants by Tzeltal children [70].

The literature identifies widespread dietary practices related to women’s reproductive stages. According to popular belief, women lose or increase “heat” in their bodies during menstruation, pregnancy, childbirth, and the postpartum period, which requires measures aimed at restoring balance and preventing the onset of other diseases [14, 29, 34, 71]. Various sources have emphasized the importance of diet in these situations [12, 19, 56, 72]. Particularly after childbirth, their body is believed to become “cold”, and its balance has to be restored through diet and sweat baths (*temazcal*) [68, 71, 73].

The descriptions of the domain of human beings contain multiple references to the belief that some individuals are “cold” or “hot” by nature, based on their gender, skin color, personality traits, and other criteria. For example, men are considered “hotter” than women [50]; a fearful and manipulative person is “cold,” while an abusive and aggressive person is “hot” [17]. The Yucatec Maya believe that some people are *síis k'ab* (cold hand) or *chokoh k'ab* (hot hand) [74, 75]. Several sources point out that this characteristic determines their aptitude for certain culinary practices, agricultural activities, and caring for domestic animals [72, 76–78]. The classification of individuals based on their nature appears to be more significant in this ethnic group than in others.

The landscape domain refers to a widely known classification pattern in Mexico. People conceive land to be divided into two major types, cold land (*tierra fría*) and hot land (*tierra caliente*). The former refers to highland areas with a temperate climate (sometimes popularly referred to as cold), and the latter refers to lower altitude areas with a warm climate [79–81]. Additionally, people recognize differences in vegetation, agricultural resources, and even cultural aspects [81, 82]. Some sources report a third landscape component, temperate land (*tierra templada*), with intermediate altitude and climate [48, 83].

This division of the landscape has rarely been linked to the hot–cold system. Some publications acknowledge this classification pattern, but as a fact separate from the classificatory system [33, 70, 84]; others mention it but do not address the system at all [81, 82]. However, there are indications that this landscape conception is part of the system. One criterion for classifying food is where it was obtained or cultivated: if it comes from hot land, it is considered “hot,” and it is “cold” if it comes from cold land [35, 68]. Maffi [85] described fertility concepts in cultivated lands, as well as implicit ideas of health and disease that fit within the hot–cold system and are associated with this landscape division. García-Hernández et al. [20] also found an apparent influence of landscape on the properties of diseases and medicinal plants.

### Geographic and cultural distribution of the hot–cold system in Mexico

**Fig. 2:**
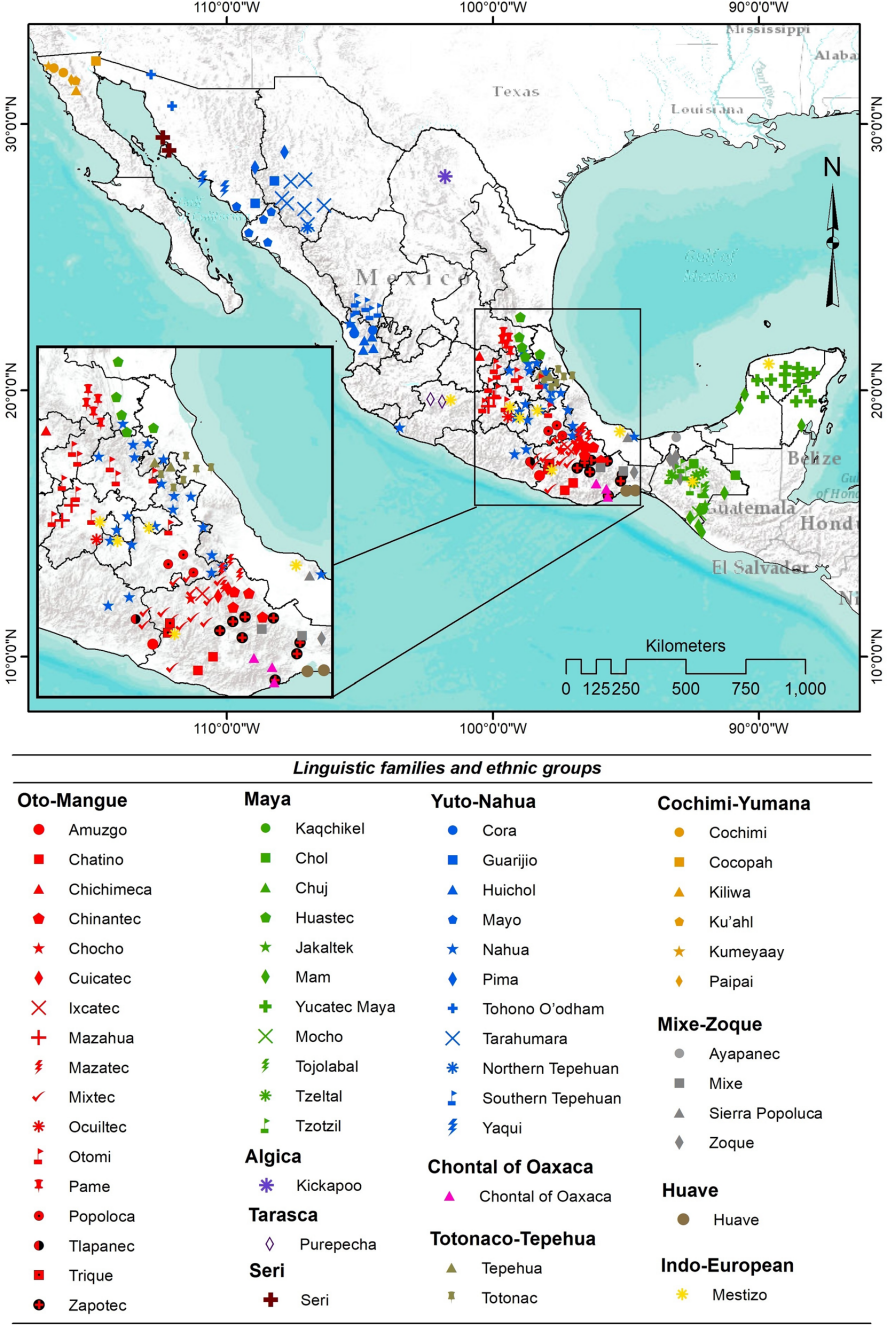
Ethnic groups and linguistic families of Mexico using the hot–cold system

The system is widely used in Mexico (Fig. 2). Of the 68 ethnic groups speaking an indigenous language, 55 have been documented to use the hot–cold system, at least in the domain of traditional medicine. With the Cochimi ethnic group (which lost its language)*,* it rises to 56, 81% of the total. All 11 indigenous language families known from Mexico are represented*.* Spanish-speaking mestizo groups and the Afro-Mexican community on the Oaxaca coast also use the hot–cold system (the latter are not shown in the map, as the sources did not identify their study locations [86]).

The best-documented ethnic groups are the Nahua (28 sources), Yucatec Maya (11), Zapotec (11), and Mixtec (10). The system has been documented in all Nahua ethnic subgroups (6) and in almost all Mixtec subgroups (9 out of 10), but only three out of the five Zapotec subdivisions (details in section H of Additional file 1).

No relevant information was found for the following populations: Matlatzincas (Oto-Mangue family), Sayula Popolucas, Oluta Popolucas, Texistepec Popolucas (Mixe-Zoque family), Lacandons, Chontal Mayas, Kanjobals, Acatecs, Quichés, Kekchís, Tekos, Awakateks, and Ixils (Maya family). Similarly, no records were found for two additional ethnic groups living in Mexico [45]: Mascogos or Maskogos (Afro-Seminole speaking descendants of black slaves from the USA who fled to Mexico in the nineteenth century), and people self-identifying as Opatas (with an extinct language). The same applies to speakers of seven indigenous languages that disappeared during the twentieth century [45]. The extinct languages can be consulted in section G of Additional file 1.

## Discussion

### Academic literature on the hot–cold system in Mexico: history

The hot–cold system has been studied mainly by two related scientific disciplines: anthropology and ethnobotany. As the system is a cultural trait, most academic publications can be expected to be anthropological, particularly from social and cultural anthropology. Given the proximity, it is also unsurprising that much early ethnographic research in Mexico was conducted by scientists from the United States, including the first works on the hot–cold system [47–49]. Various anthropologists [12, 13, 31–33, 35, 87, 88] became interested in the topic and studied it in detail, while others noted this cultural trait in their ethnographic studies [84, 89, 90], and somewhat later Mexican anthropologists also took up the subject [34, 78, 83].

Ethnobotanical research on the hot–cold system in Mexico took a different path. According to Gómez-Pompa [91], modern Mexican ethnobotany initiated in the late 1950s. Since then, a considerable part of the research has focused on the study of medicinal plants [51, 92], an area where the hot–cold classification system is highly notable. However, few studies addressed this phenomenon during the first three decades (1960–1989).

Nationality and training of the researchers may have caused the omission. During the early decades of Mexican ethnobotany, researchers and teachers were mainly biologists [93]. Their knowledge is undoubtedly necessary for the identification of botanical specimens and vegetation analysis. However, the nature of their biological training, combined with their Mexican background, may have limited their perception of cultural aspects related to the medicinal flora, such as this popular classification.

Cultural anthropologists are trained to perceive distinctive social and cultural phenomena in their own culture and in others. Thanks to foreign and Mexican anthropologists, we now have detailed descriptions of the hot–cold system that allows us to understand its expressions, functions, and symbolism in various modern human groups in Mexico. This anthropological research set the stage for other researchers to study the same topic from a different perspective.

The decline in anthropological research on the hot–cold system since the 1990s was likely due to the perception that the topic had been exhausted or sufficiently studied, along with the emergence of new research areas. Ethnobotanical publications addressing the subject have increased since that decade, coinciding with the growing number of ethnobotanical texts on Mexico [51].

### Academic literature on the hot–cold system in Mexico: main themes

The hot–cold system is prominent in two large domains: traditional medicine and food; the anthropological and ethnobotanical literature reflect this. Both domains are directly related to health, and drawing a clear boundary is difficult. However, to study them, they have to be separated, preferably with clear criteria.

We identified some themes that should be explored further, though there is incipient information. For example, are plants classified in the same way by all human groups? Are there biological characteristics of plants associated with their hot–cold properties? Are there cultural patterns of classification of the useful flora? Patterns of traditional knowledge by regions or linguistic families should be a particular focus in the future, especially for the medicinal flora. As the hot–cold system plays an underappreciated role in food preparation, composition and preferences, food is another promising subject.

The classification of human beings and landscape is not well documented, perhaps because the system is less obvious in these domains; this subject should be amenable to ethnobotanical research. The other understudied domains, such as colors, seasons, soil types, and everyday utensils, can probably be captured best through ethnographic studies.

Here, we separated the domains for analytical purposes. However, all domains form a complex framework to structure the world. Things or events that may seem unrelated have a real or symbolic relationship under the hot–cold classification system.

Finally, the hot–cold classification can have concrete applications for health professionals who deal with patients immersed in the system [94, 95]. Without adequate knowledge, they may propose treatments and dietary changes that may be rejected. For instance, women avoid consuming cold food and bathing during menstruation (a “hot” stage) for fear that the “coldness” of the food and water will damage their bodies, especially their wombs, which is believed to lead to sterility [12, 34]. For a menstruating patient, a health professional knowledgeable about these practices could then suggest foods of the appropriate property (generally “hot”) and to clean her body with as little water as possible until the end of her menstruation.

### The distribution of the hot–cold system in Mexico

The analysis shows that the system is widespread among people of different linguistic groupings, including all Indo-American language families, as well as Mestizo and Afro-Mexican groups, at least in the domain of traditional medicine. The panorama presented here is more comprehensive than the results of a previous publication [19], largely as a result of the additional information obtained from the *Indigenous Medicinal Flora of Mexico* [39] and *Traditional Medicine of Indigenous Peoples of Mexico* [40].

The apparent absence of sources on the use of the hot–cold system in some groups may be due to lack of documentation rather than its absence. Indigenous groups without records generally live very close to other ethnic groups for which the phenomenon has been documented. The Matlatzincas of San Francisco Oxtotilpan, State of Mexico, live 35 km away from San José Villa de Allende, inhabited by Mazahuas that use the system. The microregion formed by Sayula, Oluta, and Texistepec Popoluca territories in southern Veracruz is only 40 km away from the territorial core of the Sierra Popolucas, and 45 km from the Nahua community of Pajapan, both of which were recorded in this study. Kanjobals, Acatecs, Quichés, and Tekos share their area with Chujs, Mams, Kaqchikels, Jakalteks, Mochos, and Tojolabals in the southeastern part of the state of Chiapas, near Guatemala, and the hot–cold system has been documented for the latter. Kanjobals, Acatecs, Awakateks, Quichés, Kekchis, and Ixils live in the states of Campeche and Quintana Roo near Mam populations that use the system. The Chontal Mayas occupy approximately one-third of the Tabasco state's territory and are situated very close to the only community inhabited by Ayapanecs recorded in this study.

One example of the lack of documentation is the Quichés. We found no sources from Mexico for this group; however, several studies in Guatemala [96–98] confirm that they do indeed use the system. According to Felger and Moser [99], the Seri do not classify their plants or remedies as “hot” or “cold”. Indeed, neither of the two sources on this ethnic group [28, 29] contained records of the classification of medicinal plants. However, they do classify “kidney disease” as “hot,” and there are various related concepts and beliefs in their nosology [39, 40]. They seem to classify some ailments but not medicinal plants and other types of remedies. Some other ethnic groups do not explicitly classify their diseases as “hot” or “cold” despite having concepts related to the hot–cold system in their etiology or treatment [16, 34, 100]. More research is needed in these cases.

It is possible that the Lacandons are an exception, even though they live close to other groups that use the system. It was the only ethnic group for which the website *Traditional Medicine of the Indigenous Peoples of Mexico* [40] makes no reference on the hot–cold system, and none was found elsewhere. Lacandons believe their deities send them diseases for failing to comply with social norms or ritual duties; these deities may use illnesses to communicate with the sick person and express a desire [40, 101]. The absence of the system makes sense under these etiological explanations of illnesses.

According to Foster [102], non-Western medical systems mostly explain the origin of diseases based on two basic principles: personalistic and naturalistic. Personalistic systems attribute diseases to external agents, whether human (witches, sorcerers), nonhuman (ghosts, spirits, ancestors), or supernatural (deities and similar entities), often in response to human misbehavior. In contrast, naturalistic systems essentially conceive diseases as a result of bodily imbalance or an imbalance between the sick individual and their social or natural environment; that is, they are attributed to natural causes. Explanations of diseases under the hot–cold system belong to a naturalistic system. Foster [102] specified that both naturalistic and personalistic systems can coexist in a society, but one usually predominates. Lacandons appear to have a predominantly personalistic etiology, which could explain the apparent absence of the system.

There are indications that the system has varying levels of relevance in the traditional medicine of Mexico. The system is undoubtedly important for Mixtecs [62, 68, 71, 100], Chochos [16], Zapotecs [13, 15, 32], Otomis [18], Nahuas [35, 103, 104], and Zoques [36, 63]. In these cases, the hot–cold system is applied to both diseases and medicinal plants. In Yucatec Maya communities, the system is relevant for explaining their diseases, but it has less importance in their medicinal flora [55]. In traditional Mixe medicine, the system has little relevance in both the etiology of diseases and medicinal plants [52, 53].

The documentation of the hot–cold system has concentrated on the Nahua, Yucatec Maya, Zapotec and Mixtec ethnic groups, the most numerous [105], widespread and studied peoples. However, there are some large geographical gaps: parts of western and northern Mexico, the Gulf coast and some regions of the Yucatán peninsula. The first three regions have relatively few indigenous peoples and therefore have been less interesting to anthropologists and ethnobotanists. However, the lack of information from some areas of the Yucatán Peninsula is striking, and we have no explanation.

This study provides a general overview of the use of the hot–cold system by different ethnic groups in Mexico. To interpret it correctly, some issues related to the ethnicity of the groups and the temporality of the sources must be considered.

First, language was used as a somewhat flexible ethnic marker in this study. For example, Mixe language speakers were considered Mixes, but self-identification was also considered. “Mestizo” essentially refers to individuals whose primary or sole language is Spanish, without self-identifying as indigenous. But in most cases, mestizos have indigenous ancestry, resulting in indigenous cultural influences on mestizo culture even in urban populations, as indicated in section B of the Additional file 1.

Second, our sources span approximately a century. Language and local culture today may differ from the original descriptions. However, it appears that despite sociocultural changes, certain characteristics, such as the hot–cold system, may persist. George Foster visited Tzintzuntzan repeatedly for over 3 decades (approximately from 1958 to 1992) and observed that, despite the passage of time, the hot–cold system remained in effect:

“…In Tzintzuntzan I find that, in spite of the general acceptance of modern biomedicine for most medical problems, an astonishingly high percentage of illness episodes, including most of those treated by physicians, is explained pos facto in terms of hot and cold experiences” [50].

Even in present-day large cities, people widely apply the hot–cold system, as is obvious even to a casual observer. Recent studies of patients treated in healthcare centers in major cities in Mexico have shown that they apply the hot–cold classification system to both diseases and medicinal plants [106, 107]. These studies do not specify the ethnicity of the patients, but presumably the majority are assimilated into urban lifestyles.

The hot–cold system is spread across an ethnic and cultural continuum, represented on one end by monolingual speakers of indigenous languages living in rural communities and on the other by urban individuals who use Spanish as their only or main language. Historian Alfredo López Austin aptly illustrated this matter:

“La herencia cultural no se limita a los descendientes indígenas de los mesoamericanos, pues en los más diversos segmentos de la sociedad mexicana puede encontrarse, entre muchas otras creencias y prácticas, la división de enfermedades, medicinas y alimentos separados en fríos y calientes. Y no son sólo pautas clasificatorias sino guías de acciones dirigidas a conservar o recuperar la salud” [3].

[“Cultural heritage is not limited to the indigenous descendants of Mesoamericans, as among the most diverse segments of Mexican society, one can find, among many other beliefs and practices, the division of illnesses, medicines, and foods into cold and hot. And these are not just classificatory guidelines, but also guiding principles for actions aimed at preserving or restoring health” [3].]

The current hot–cold system is present in ethnic groups outside the Mesoamerican cultural area. This raises two questions: how and when did it spread? We suggest the possibility that the syncretism of European and Native American ideas took place in the central Mesoamerican area and then spread northward. This would explain the presence of hot–cold concepts of illnesses of the Seris but not in their remedies, indicating that perhaps only a part of the system reached them. It would also explain the observations of Latorre and Latorre [59], who apparently noted that the hot–cold system is not originally part of Kickapoo culture: “The Kickapoos have adopted many of the Mexican beliefs concerned with heat and cold, whether in food, beverages, medicine, or the state of the body.” Future studies of the cultural evolution and recent history (last 500 years) of living and extinct ethnic groups in Mexico could resolve these questions.

## Conclusions

The hot–cold system in Mexico has been documented for approximately a century. Though anthropologists laid the foundations, ethnobotanists appear to have a growing interest. Traditional medicine and food are the best-documented domains. However, the large amount of information currently available needs to be integrated and analyzed to design field studies aimed at specific questions. The domain of the landscape is particularly interesting since it is closely linked to the classification of useful flora. The system applies to many domains, and they are all interconnected in everyday life.

The system is extensively distributed in Mexico and is used by the majority of indigenous groups in Mexico, as well as Mestizo and Afro-Mexican groups; therefore, it is an important cultural heritage of the country. An interesting, unresolved question is how the system spread between and among ethnic groups, if it is absent in some groups and why.

The hot–cold classification and its associated worldview is alive and has applications and consequences for the health, nutrition and well-being of many groups in Mexico, including urban people using contemporary services. Most of the professionals who attend to their needs do not know how to address the issue and just ignore it. This research has concrete practical applications.

### Supplementary Information

**Additional file 1**. Consulted sources and information obtained.

## Data Availability

All data generated or analyzed are included in this published article.
