# Supplementary material for: Academic history, domains and distribution of the hot-cold system in Mexico
Source: J Ethnobiol Ethnomed. 2023 Nov 2;19:50. doi: 10.1186/s13002-023-00624-1 (PMC10623800; doi:10.1186/s13002-023-00624-1)
Supplement: Supplementary file 1 — Additional file 1. Consulted sources and information obtained. [file 13002_2023_624_MOESM1_ESM.docx]

**García-Hernández et al. Academic history, domains and distribution of the hot–cold system in Mexico**

**Supplementary Information**

**CONSULTED SOURCES AND OBTAINED INFORMATION**

**Index**

[Section A. List of sources on the use of the hot-cold system by different human groups and locations of Mexico 2](#_30j0zll)

[Section B. Information on the scholarly publications 11](#_1fob9te)

[Section C. Information on the printed monographs 19](#_3znysh7)

[Section D. Information on the sections of the web site “Atlas de los Pueblos Indígenas de México” (http://atlas.inpi.gob.mx) 20](#_2et92p0)

[Section E. Information on the sections of the web page "Flora Medicinal Indígena de México" (http://www.medicinatradicionalmexicana.unam.mx/fmim/index.html) 21](#_tyjcwt)

[Section F. Information on the sections of the web page "La Medicina Tradicional de los Pueblos Indígenas de México" (http://www.medicinatradicionalmexicana.unam.mx/mtpim/index.html) 24](#_3dy6vkm)

[Section G. List of linguistic and ethnic groups of Mexico using the hot-cold system 30](#_1t3h5sf)

[Section H. List of Nahua, Zapotec and Mixtec ethnic groups using the hot-cold system 34](#_4d34og8)

# Section A. List of sources on the use of the hot-cold system by different human groups and locations of Mexico

**Scholarly publications**

1. Acuña AM, Caso L, Aliphat MM, Vergara CH. Edible insects as part of the traditional food system of the Popoloca town of Los Reyes Metzontla, Mexico. J Ethnobio. 2011; doi:10.2993/0278-0771-31.1.150.

2. Alberti-Manzanares P. Los aportes de las mujeres rurales al conocimiento de plantas medicinales en México: Análisis de género. Agricultura, Sociedad y Desarrollo. 2006;3:139–53.

3. Alcorn JB. Huastec Mayan Ethnobotany. Austin: University of Texas Press; 1984.

4. Álvarez Heydenreich L. La enfermedad y la cosmovisión en Hueyapan, Morelos. México, D.F.: Instituto Nacional Indigenista; 1987.

5. Álvarez Heydenreich L. Breve estudio de las plantas medicinales en Hueyapan, Morelos. In: Viesca Treviño C, editor. Estudios sobre etnobotánica y antropología médica. México, D.F.: Instituto Mexicano para el Estudio de las Plantas Medicinales; 1976. p. 85–111.

6. Álvarez-Quiroz V, Caso-Barrera L, Aliphat-Fernández M, Galmiche-Tejeda A. Plantas medicinales con propiedades frías y calientes en la cultura zoque de Ayapa, Tabasco, México. Bol Latinoam Caribe Plantas Med Aromat. 2017;16:428–54.

7. Anderson EN, Cahuich Canal J, Dzib A, Flores Guido S, Islebe G, Medina Tzuc F, et al. Las plantas de los mayas: etnobotánica en Quintana Roo, México. San Cristóbal de las Casas: El Colegio de la Frontera Sur; 2005.

8. Ankli A, Sticher O, Heinrich M. Medical ethnobotany of the Yucatec Maya: healers’ consensus as a quantitative criterion. Econ Bot. 1999; doi:10.1007/BF02866493.

9. Ankli A, Sticher O, Heinrich M. Yucatec Maya medicinal plants versus nonmedicinal plants: Indigenous characterization and selection. Hum Ecol. 1999; doi:10.1023/A:1018791927215.

10. Aparicio Aparicio JC, Voeks RA, Funch LS. Mixtec taxonomy: Plant classification, nomenclature, and identification in Oaxaca, Mexico. Ethnobot Res App. 2021; doi:10.32859/era.21.24.1-13.

11. Báez L. Saberes y prácticas terapéuticas entre los nahuas de Naupan, Puebla. In: Gallardo Arias P, editor. Curanderos y medicina tradicional en la Huasteca. México: Ediciones del Programa de Desarrollo Cultural de la Huasteca; 2008. p. 209–37.

12. Bayles B. Metaphors to cure by: Tojolab’al Maya midwifery and cognition. Anthopol Med. 2008; doi:10.1080/13648470802357554.

13. Bonfil Batalla G. Diagnóstico sobre el hambre en Sudzal, Yucatán: un ensayo de antropología aplicada. México, D.F.: Centro de Investigaciones y Estudios Superiores en Antropología Social, Universidad Autónoma Metropolitana, Universidad Iberoamericana; 2006.

14. Boster JS, Weller SC. Cognitive and contextual variation in hot-cold classification. Am Anthropol. 1990; doi:10.1525/aa.1990.92.1.02a00120.

15. Browner CH. Criteria for selecting herbal remedies. Ethnology. 1985; doi:10.2307/3773487.

16. Camacho-Hernández C, Lagunez-Rivera L, Aguilar-Contreras A, Solano Gómez R. Ethnobotany of medicinal flora in two communities of the Mixteca Alta in Oaxaca, Mexico. Bot Sci. 2022; doi:10.17129/botsci.2947.

17. Casas A, Viveros JL, Katz E, Caballero J. Las plantas en la alimentación mixteca: una aproximación etnobotánica. Am Indig. 1987;47:317–43.

18. Castro R. La lógica de una de las creencias tradicionales en salud: Eclipse y embarazo en Ocuituco, México. Salud Publica Mex. 1995;37:329–38.

19. Cervantes M, Zapién G. El mito de los alimentos fríos o calientes y su realidad nutricional. Narrativas Antropológicas. 2020;9–18.

20. Chevalier JM, Sánchez Bain A. The hot and the cold: ills of humans and maize in native Mexico. Toronto: University of Toronto Press; 2003.

21. Currier RL. The hot-cold syndrome and symbolic balance in Mexican and Spanish-American folk medicine. Ethnology; 1966; doi:10.2307/3772771.

22. De la Fuente Chicoséin J. Yalálag: una villa zapoteca serrana. 2nd ed. México, D.F.: Comisión Nacional para el Desarrollo de los Pueblos Indígenas; 2012.

23. Foster GM. Hippocrates’ Latin American legacy: humoral medicine in the New World. Langhorne: Gordon and Breach; 1994.

24. Foster GM. The validating role of humoral theory in traditional Spanish-American therapeutics. Am Ethnol. 1988; doi:10.1525/ae.1988.15.1.02a00080.

25. Foster GM. How to get well in Tzintzuntzan. Soc Sci Med. 1985; doi:10.1016/0277-9536(85)90129-7.

26. Foster GM. How to stay well in Tzintzuntzan. Soc Sci Med. 1984; doi:10.1016/0277-9536(84)90048-0.

27. Foster GM. The concept of ‘neutral’ in humoral medical systems. Med Anthropol: Cross Cult Stud Health Illn. 1984; doi:10.1080/01459740.1984.9965902.

28. Foster GM. Methodological problems in the study of intracultural variation: the hot/cold dichotomy in Tzintzuntzan. Hum Org. 1979; doi:10.17730/humo.38.2.n36u8p1453m06v34.

29. Foster GM. Tzintzuntzan: los campesinos en un mundo en cambio. México, D.F: Fondo de Cultura Económica; 1972.

30. Foster GM. Empire’s children: the people of Tzintzuntzan. Mexico: Smithsonian Institution, Institute of Social Anthopology; 1948.

31. Foster GM. The geographical, linguistic, and cultural position of the Popoluca of Veracruz. Am Anthropol. 1943;45:531–46.

32. Frei B, Sticher O, Viesca C, Heinrich M. Medicinal and food plants: Isthmus Sierra Zapotec criteria for selection. J Appl Bot Food Qual. 1998;72:82–6.

33. Furbee L, Benfer RA. Cognitive and geographic maps: study of individual variation among Tojolabal Mayans. Am Anthropol. 1983; doi:10.1525/aa.1983.85.2.02a00030.

34. García-Hernández KY, Vibrans H, Rivas-Guevara M, Aguilar-Contreras A. This plant treats that illness? The hot–cold system and therapeutic procedures mediate medicinal plant use in San Miguel Tulancingo, Oaxaca, Mexico. J Ethnopharmacol. 2015; doi:10.1016/j.jep.2015.01.001.

35. Geck MS, Cabras S, Casu L, Reyes García AJ, Leonti M. The taste of heat: how humoral qualities act as a cultural filter for chemosensory properties guiding herbal medicine. J Ethnopharmacol. 2017; doi:10.1016/j.jep.2017.01.027.

36. Geck MS, Reyes García AJ, Casu L, Leonti M. Acculturation and ethnomedicine: a regional comparison of medicinal plant knowledge among the Zoque of southern Mexico. J Ethnopharmacol. 2016; doi:10.1016/j.jep.2016.04.036.

37. Giovannini P, Heinrich M. Xki yoma’ (our medicine) and xki tienda (patent medicine)—Interface between traditional and modern medicine among the Mazatecs of Oaxaca, Mexico. J Ethnopharmacol. 2009; doi:10.1016/j.jep.2008.11.003.

38. Groark KP. Vital warmth and well-being: steambathing as household therapy among the Tzeltal and Tzotzil Maya of highland Chiapas, Mexico. Soc Sci Med. 2005; doi:10.1016/j.socscimed.2004.08.044.

39. Heinrich M. Indigenous concepts of medicinal plants in Oaxaca: lowland Mixe plant classification based on organoleptic characteristics. J Appl Bot Food Qual. 1998;72:75–81.

40. Heinrich M, Rimpler H, Barrera NA. Indigenous phytotherapy of gastrointestinal disorders in a lowland Mixe community (Oaxaca, Mexico): Ethnopharmacologic evaluation. J Ethnopharmacol. 1992; doi:10.1016/0378-8741(92)90062-V.

41. Holland WR. Medicina maya en los Altos de Chiapas: un estudio del cambio socio-cultural. México, D.F: Instituto Nacional Indigenista; 1990.

42. Ingham JM. On Mexican folk medicine. Am Anthropol. 1970; doi:10.1525/aa.1970.72.1.02a00090.

43. Jorand B. Formas de transformación del conocimiento de la medicina tradicional en los pueblos nahuas del municipio de Hueyapan, Sierra Norte de Puebla. Cuicuilco Rev cienc antropol. 2008;15:181–96.

44. Kaplan LN, Kaplan L. Medicinal plant and food use as related to health and disease in coastal Oaxaca. In: Wallace AFC, editor. Selected papers of the Fifth International Congress of Anthropological and Ethnological Sciences. Philadelphia: University of Pennsylvania; 1956. p. 452–8.

45. Katz E. Vapor, aires y serpientes: meteorología en la “Tierra de la Lluvia” (Mixteca Alta, Oaxaca). In: Lammel A, Goloubinoff M, Katz E, editors. Aires y lluvias: antropología del clima en México. México, D.F.: Centro de Investigaciones y Estudios Superiores en Antropología Social, Centro de Estudios Mexicanos y Centroamericanos, Institut de Recherche pour le Développement; 2008. p. 283–322.

46. Katz E. Recovering after childbirth in the Mixtec highlands (Mexico). In: Schroeder E, Balansard G, Cabalion P, Fleurentin J, Mazars G, editors. Medicines and foods: the ethnopharmacogical approach. Paris: ORSTOM Éditions, Société Française d’Ethnopharmacologie; 1996. p. 97–109.

47. Katz E. El papel de la etnobiología en el estudio de los sistemas de producción agrícola: el ejemplo de una zona cafetalera de la Mixteca Alta. In: Navarra Garza H, Colin J-P, Milleville P, editors. Sistemas de producción y desarrollo Agrícola. México, D.F.: Colegio de Postgraduados, Instituto Francés de Investigación Científica para el Desarrollo en Cooperación (ORSTOM); 1993. p. 321–7.

48. Katz E. El temazcal: entre religión y medicina. In: Dahlgren Jordan B, editor. III Coloquio de historia de la religión en Mesomérica y áreas afines. México, D.F.: Universidad Nacional Autónoma de México; 1993. p. 175–85.

49. Katz E. Del frío al exceso de calor: dieta alimenticia y salud en la Mixteca. In: Sesia P, editor. Medicina tradicional, herbolaria y salud comunitaria en Oaxaca. Oaxaca: Centro de Investigaciones y Estudios Superiores en Antropología Social, Gobierno del Estado de Oaxaca; 1992. p. 99–113.

50. Katz E. De los mixtecos: medio ambiente y grupos sociales. Trace. 1991;51–4.

51. Katz E, Vargas Guadarrama LA. Cambio y continuidad en la alimentación de los mixtecos. An Antropol. 1990;27:15–51.

52. Kay M, Yoder M. Hot and cold in women’s ethnotherapeutics: the American-Mexican west. Soc Sci Med. 1987; doi:10.1016/0277-9536(87)90273-5.

53. Kearney M. Los vientos de Ixtepeji: concepción del mundo y estructura social de un pueblo zapoteco. México D.F.: Instituto Indigenista Americano; 1971.

54. Latorre FA, Latorre DL. The Mexican Kickapoo indians. Austin: University of Texas Press; 1976.

55. Le Guen O. Temperature terms and their meaning in Yucatec Maya (Mexico). In: Koptjevskaja-Tamm M, editor. The linguistics of temperature. Amsterdam: John Benjamins Publishing Company; 2015. p. 742–75.

56. Leonti M, Sticher O, Heinrich M. Medicinal plants of the Popoluca, México: organoleptic properties as indigenous selection criteria. J Ethnopharmacol. 2002; doi:10.1016/S0378-8741(02)00078-8.

57. Leonti M, Vibrans H, Sticher O, Heinrich M. Ethnopharmacology of the Popoluca, Mexico: an evaluation. J Pharm Pharmacol. 2001; doi:10.1211/0022357011778052.

58. Letcher Lazo CJ. La mano fría y la mano caliente: un estudio de la metonimia en el maya yucateco. Estud Cult Maya. 2022; doi:10.19130/iifl.ecm.59.22X878.

59. Lewis O. Tepoztlán: un pueblo de México. México D.F.: Editorial Joaquín Mortiz; 1968.

60. Lewis O. Life in a Mexican village: Tepoztlán restudied. Urbana: University of Illinois Press; 1951.

61. López Hernández JR, Teodoro Méndez JM. La cosmovisión indígena tzotzil y tzetzal a través de la relación salud-enfermedad en el contexto de la medicina tradicional indígena. Ra Ximhai. 2006;2:15–26.

62. Lorente Fernández D. El «frío» y el «calor» en el sistema médico nahua de la sierra de Texcoco: una aproximación. Rev Esp Antropol Am. 2012;42:251–60.

63. Madsen W. Hot and cold in the universe of San Francisco Tecospa, Valley of Mexico. J Am Folk. 1955; doi:10.2307/537248.

64. Maffi L. Domesticated land, warm and cold: linguistic and historical evidence on Tenejapa Tzeltal Mayan ethnoecology. In: Gragson TL, Blount BG, editors. Ethnoecology: knowledge, resources, and rights. Athens: University of Georgia Press; 1999. p. 41–56.

65. Mak C. Mixtec medical beliefs and practices. Am Indig. 1959;19:125–50.

66. Martínez Spinoso MY, Miranda Perkins K. Etnobotánica de los xi’ui de la Sierra Gorda de Querétaro. In: Gallardo Arias P, editor. Curanderos y medicina tradicional en la Huasteca. México: Programa de Desarrollo Cultural de la Huasteca; 2008. p. 123–73.

67. Mathews HF. Context-specific variation in humoral classification. Am Anthropol. 1983; doi:10.1525/aa.1983.85.4.02a00050.

68. McCullough JM. Human ecology, heat adaptation, and belief systems: the hot-cold syndrome of Yucatan. J Anthropol Res. 1973; doi:10.1086/jar.29.1.3629623.

69. Messer E. Systematic and medicinal reasoning in Mitla folk botany. J Ethnopharmacol. 1991; doi:10.1016/0378-8741(91)90170-I.

70. Messer E. The hot and cold in Mesoamerican indigenous and Hispanicized thought. Soc Sci Med. 1987; doi:10.1016/0277-9536(87)90272-3.

71. Messer E. Hot-cold classification: theoretical and practical implications of a Mexican study. Soc Sci Med. 1981; doi:10.1016/0160-7987(81)90036-3.

72. Messer E. Zapotec plant knowledge: classification, uses and communications about plants in Mitla, Oaxaca, México. In: Flannery KV, Blanton RE, editors. The vegetational history of the Oaxaca Valley and Zapotec plant knowledge. Ann Arbor: Museum of Anthropology, University of Michigan; 1978. pp. 6-198.

73. Molony CH. Systematic valence coding of Mexican “hot"—"cold” food. Ecol Food Nutr. 1975; doi:10.1080/03670244.1975.9990411.

74. Mondragón D, Villa-Guzmán DM. Estudio etnobotánico de las bromelias epífitas en la comunidad de Santa Catarina Ixtepeji, Oaxaca, México. Polibotanica. 2008;175–91.

75. Nash J. The logic of behavior: curing in a Maya Indian town. Hum Org. 1967;26:132–40.

76. Olavarrieta Marenco M. Magia en los Tuxtlas, Veracruz. México, D.F.: Consejo Nacional para la Cultura y las Artes, Instituto Nacional Indigenista; 1977.

77. Ortiz de Montellano BR, Browner CH. Chemical bases for medicinal plant use in Oaxaca, Mexico. J Ethnopharmacol. 1985; doi:10.1016/0378-8741(85)90062-5.

78. Petrich P. La alimentación mochó: acto y palabra (estudio etnolingüístico). San Cristóbal de las Casas: Universidad Autónoma de Chiapas; 1985.

79. Pulido Secundino J, Bocco Verdinelli G. Conocimiento tradicional del paisaje en una comunidad indígena: caso de estudio en la región purépecha, occidente de México. Investigaciones Geográficas. 2016; doi:10.14350/rig.46478.

80. Quiroz Guerrero I, Pérez Vázquez A. Knowledge and preference of medicinal plants in three rural communities of Tezonapa, Veracruz, Mexico. Glob Adv Res JMed Med Sci. 2016;5:123–9.

81. Ramírez Torres JL. Del binomio frío-caliente a la complejidad poliédrica de una estructura mesoamericana completa. Toluca: Ediciones Eón, Universidad Autónoma del Estado de México; 2017.

82. Redfield R. The folk culture of Yucatan. Chicago: The University of Chicago Press; 1941.

83. Redfield R. Tepoztlán: a Mexican village: A study of folk life. Chicago: The University of Chicago Press; 1930.

84. Redfield R, Villa Rojas A. Chan Kom: a Maya village. Chicago: The University of Chicago Press; 1962.

85. Rojas-Alba M. Clasificación tradicional de los alimentos frío-caliente en un pueblo de origen náhuatl. Tlahui-Medic. 1996. http://www.tlahui.com/friocal1.html. Accessed 16 Jan 2023.

86. Ryesky D. Conceptos tradicionales de la medicina en un pueblo mexicano: un análisis antropológico. México, D.F: Secretaría de Educación Pública; 1976.

87. Sánchez Bonilla DA. Santa Catarina del Monte: patrimonio vivo entre música y flores. Saarbrücken: Editorial Académica Española; 2012.

88. Sánchez-González A, Granados-Sánchez D, Simón-Nabor R. Uso medicinal de las plantas por los otomíes del municipio de Nicolás Flores, Hidalgo, México. Rev Chapingo Ser Hortic. 2008;14:271–9.

89. Signorini I. Patterns of fright: multiple concepts of susto in a Nahua-Ladino community of the Sierra de Puebla (Mexico). Ethnology. 1982; doi:10.2307/3773762.

90. Signorini I. Los huaves de San Mateo del Mar. México, D.F: Instituto Nacional Indigenista; 1979.

91. Silver DB. Enfermedad y curación en Zinacantán: esquema provisional. In: Vogt EZ, editor. Los zinacantecos. México: Instituto Nacional Indigenista; 1966. p. 455–73.

92. Smith-Oka V. An analysis of two indigenous reproductive health illnesses in a Nahua community in Veracruz, Mexico. J Ethnobiol Ethnomed. 2012; doi:10.1186/1746-4269-8-33.

93. Smith-Oka V. Plants used for reproductive health by Nahua women in northern Veracruz, Mexico. Econ Bot. 2008; doi:10.1007/s12231-008-9026-7.

94. Solís-Becerra CG, Estrada-Lugo EIJ. Prácticas culinarias y (re)conocimiento de la diversidad local de verduras silvestres en el Colectivo Mujeres y Maíz de Teopisca, Chiapas, México. LiminaR, Estudios Sociales y Humanísticos. 2014; doi:10.29043/liminar.v12i2.348.

95. Tiedje K. Curación y maleficio entre los nahuas potosinos. In: Gallardo Arias P, editor. Curanderos y medicina tradicional en la Huasteca. México: Programa de Desarrollo Cultural de la Huasteca; 2008. p. 17–54.

96. Torres Méndez SA, Caso Barrera L, Aliphat Fernández MM. Conocimiento ecológico, alimentación tradicional y clasificación frío-caliente: la perspectiva de los niños tseltales de Tenejapa, Chiapas. LiminaR Estudios Sociales y Humanísticos. 2019; doi:10.29043/liminar.v17i2.673.

97. Vázquez Medina B, Martínez Corona B, Aliphat Fernández MM, Aguilar Contreras A. Uso y conocimiento de plantas medicinales por hombres y mujeres en dos localidades indígenas en Coyomeapan, Puebla, México. Interciencia. 2011;36:493–9.

98. Villa Rojas A. Terapéutica tradicional y medicina moderna entre los mayas de Yucatán. An Antropol. 1981; doi:10.22201/iia.24486221e.1981.2.24118.

99. Weimann C, Heinrich M. Concepts of medicinal plants among the Nahua of the Sierra de Zongolica, Veracruz (Mexico). J Appl Bot Food Qual. 1998;72:87–91.

100. Young JC. Medical choice in a Mexican village. New Brunswick: Rutgers University Press; 1981.

101. Young JC. Illness categories and action strategies in a Tarascan town. Am Ethnol. 1978; doi:10.1525/ae.1978.5.1.02a00070.

**Printed monographs**

102. Gámez Espinosa A. Popolocas. México, D.F: Comisión Nacional para el Desarrollo de los Pueblos Indígenas; 2006.

103. Quintana Hernández F, Luis Rosales C. Mames de Chiapas. México, D.F: Comisión Nacional para el Desarrollo de los Pueblos Indígenas; 2006

104. Torres Cisneros G. Mixes. México, D.F: Comisión Nacional para el Desarrollo de los Pueblos Indígenas, Programa de las Naciones Unidas para el Desarrollo; 2004.

105. Valle Esquivel J. Nahuas de la Huasteca. México, D.F.: Comisión Nacional para el Desarrollo de los Pueblos Indígenas, Programa de las Naciones Unidas para el Desarrollo; 2004.

**Web pages**

106. Instituto Nacional de los Pueblos Indígenas-Instituto Nacional de Lenguas Indígenas. Atlas de los pueblos indígenas de México. http://atlas.inpi.gob.mx/ (2020). Accessed 25 Jan 2023.

107. Universidad Nacional Autónoma de México. Flora medicinal indígena de México. http://www.medicinatradicionalmexicana.unam.mx/fmim/index.html (2009). Accessed 25 Jan 2023.

108. Universidad Nacional Autónoma de México. La medicina tradicional de los pueblos indígenas de México. http://www.medicinatradicionalmexicana.unam.mx/mtpim/index.html (2009). Accessed 25 Jan 2023.

# Section B. Information on the scholarly publications

Publication number (# pub) in correspondence with the list in section A. Type: J = journal paper; B = book; C = chapter in book; P = proceedings of an academic meeting. In-depth study: Y = yes, it explores the cold-hot system or at least shows some particularities; N = not, it only mentions the use of the system. Approach: An=anthropological; Eb=ethnobotanical; Ez=ethnozoological; Ee=ethnoecological; S=social. Domain(s): TM = traditional medicine; F = food; L = landscape; Pe = people; O = others. (NS) = Not specified. Superscripts indicate comments at the table footnotes. The details of the classification and nomenclature of the ethnic groups, languages and language families can be found in the sections G and H. The numbers in parentheses specify corresponding localities, municipalities, states, ethnic groups, linguistic groups and language families. For example, in publication 3, the municipalities Tanlajás, San Antonio, Tancahuitz, Aquismón, Tampamolón, Tanquián de Escobedo, Huehuetlán, Ciudad Valles (1) correspond to the state San Luis Potosí (1), and the municipality Tantoyuca (2) is located in the state of Veracruz de Ignacion de la Llave (2)

| **Publication details** | | | | | | **Data of the study area** | | | | | |
| --- | --- | --- | --- | --- | --- | --- | --- | --- | --- | --- | --- |
| **# pub** | **Year** | **Type** | **In- depth study** | **Approach** | **Do-**  **main(s)** | **Locality(ies)** | **Municipality(ies)** | **State(s)** | **Ethnic group(s) or subgroup(s)** | **Linguistic group(s)** | **Linguistic family(ies)** |
| 1 | 2011 | J | Y | Ez | F | Los Reyes Metzontla | Zapotitlán | Puebla | Popoloca | Popoloca | Oto-Mangue |
| 2 | 2006 | J | N | S | TM | (NS) | Natívitas | Tlaxcala | Mestizo^1,2^ | Nahuatl | Yuto-Nahua |
| 3 | 1994 | B | Y | Eb | TM | 22 of San Luis Potosí and 2 of Veracruz de Ignacio de la Llave (NS) | Tanlajás, San Antonio, Tancahuitz, Aquismón, Tampamolón, Tanquián de Escobedo, Huehuetlán, Ciudad Valles (1);  Tantoyuca (2) | San Luis Potosí (1); Veracruz de Ignacio de la Llave (2) | Huastec | Huastec | Maya |
| 4 | 1976 | C | Y | An | TM, F | Hueyapan (San Andrés Hueyapan) | Hueyapan | Morelos | Center Nahua | Nahuatl | Yuto-Nahua |
| 5 | 1987 | B | Y | An | TM, F, Pe, L, O | Hueyapan (San Andrés Hueyapan) | Hueyapan | Morelos | Center Nahua | Nahuatl | Yuto-Nahua |
| 6 | 2017 | J | Y | Eb | TM | Ayapa | Jalpa de Méndez | Tabasco | Ayapanec | Ayapanec | Mixe-Zoque |
| 7 | 2005 | B | N | Eb | TM, F | Chunhuhub (and surroundings) | Felipe Carrillo Puerto | Quintana Roo | Yucatec Maya | Yucatec | Maya |
| 8 | 1999 | J | Y | Eb | TM | Chikindzonot (1); Ekpedz (2);  X-Cocmil (3) | Chikindzonot (1); Tixcacalcupul (2); Tekom (3) | Yucatán | Yucatec Maya | Yucatec | Maya |
| 9 | 1999 | J | Y | Eb | TM | Chikindzonot (1); Ekpedz (2);  X-Cocmil (3) | Chikindzonot (1); Tixcacalcupul (2); Tekom (3) | Yucatán | Yucatec Maya | Yucatec | Maya |
| 10 | 2019 | J | N | Eb | TM, F | General Lázaro Cárdenas, Villa de Guadalupe Victoria, Miguel Hidalgo, Iturbide, Morelos, Madero, Vicente Guerrero, Ignacio Zaragoza, Benito Juárez | San Miguel el Grande | Oaxaca | Mixtec of the central region (east of Putla) | Mixtec | Oto-Mangue |
| 11 | 2008 | C | Y | An | TM | (NS) | Naupan | Puebla | Northeastern Nahua | Nahuatl | Yuto-Nahua |
| 12 | 2008 | J | Y | An | TM, F, Pe, O | (NS) | (NS) | Chiapas | Tojolabal | Tojolabal | Maya |
| 13 | 1962^3^ | B | Y | An | F, Pe | Sudzal | Sudzal | Yucatán | Yucatec Maya | Yucatec | Maya |
| 14 | 1990 | J | Y | An | F | (NS) | (NS) | Tlaxcala | (NS) | (NS) | (NS) |
| 15 | 1985 | J | Y | Eb | TM | (NS) | (NS) | Oaxaca | Chinantec | Chinantec | Oto-Mangue |
| 16 | 2022 | J | Y | Eb | TM | San Andrés Nuxiño (1);  San Pedro Topiltepec (2) | San Andrés  Nuxiño (1);  San Pedro Topiltepec (2) | Oaxaca | Eastern Mixtec (1); Mixtec of the central region (southeast of Tlaxiaco) (2) | Mixtec | Oto-Mangue |
| 17 | 1987 | J | Y | Eb | F | Alcozauca de Guerrero, San José Lagunas (Capulín), Amapilca, Ixcuinatoyac (1); San Pedro Yosotatu (2) | Alcozauca de Guerrero (1); Heroica Ciudad de  Tlaxiaco (2) | Guerrero (1); Oaxaca (2) | Mixtec of northernGuerrero (1), Mestizo^2^ (2) | Mixtec | Oto-Mangue |
| 18 | 1995 | J | Y | An | TM, O | Ocuituco | Ocuituco | Morelos | Mestizo^2^ | Nahuatl | Yuto-Nahua |
| 19 | 2020 | J | Y | An | F | Coatetelco | Coatetelco | Morelos | Center Nahua | Nahuatl | Yuto-Nahua |
| 20 | 2003 | B | Y | An | TM, F, Pa, O | Pajapan (1); Soteapan (2) | Pajapan (1); Soteapan (2) | Veracruz de Ignacio de la Llave | Southern Nahua (1); Sierra Popoluca (2) | Nahuatl (1), Sierra Popoluca (2) | Yuto-Nahua, Mixe-Zoque |
| 21 | 1966 | J | Y | An | TM, F | Erongarícuaro | Eronguarícuaro | Michoacán de Ocampo | Mestizo^2^ | Tarascan | Tarasca |
| 22 | 1949^3^ | B | Y | An | TM, F, L, O | Villa Hidalgo (and others) | Villa Hidalgo | Oaxaca | Zapotec of the Sierra Juarez | Zapotec | Oto-Mangue |
| 23 | 1994 | B | Y | An | TM, F, Pe, O | Tzintzuntzan | Tzintzuntzan | Michoacán de Ocampo | Purepecha | Tarascan | Tarasca |
| 24 | 1988 | J | Y | An | TM | Tzintzuntzan | Tzintzuntzan | Michoacán de Ocampo | Mestizo^2^ | Tarascan | Tarasca |
| 25 | 1985 | J | Y | An | TM, F | Tzintzuntzan | Tzintzuntzan | Michoacán de Ocampo | Mestizo^2^ | Tarascan | Tarasca |
| 26 | 1984 | J | Y | An | TM, F, Pe | Tzintzuntzan | Tzintzuntzan | Michoacán de Ocampo | Mestizo^2^ | Tarascan | Tarasca |
| 27 | 1984 | J | Y | An | TM, F | Tzintzuntzan | Tzintzuntzan | Michoacán de Ocampo | Mestizo^2^ | Tarascan | Tarasca |
| 28 | 1979 | J | Y | An | TM, F | Tzintzuntzan | Tzintzuntzan | Michoacán de Ocampo | Mestizo^2^ | Tarascan | Tarasca |
| 29 | 1967^3^ | B | Y | An | TM, F | Tzintzuntzan | Tzintzuntzan | Michoacán de Ocampo | Mestizo^2^ | Tarascan | Tarasca |
| 30 | 1948 | B | N | An | F, L, O | Tzintzuntzan | Tzintzuntzan | Michoacán de Ocampo | Mestizo^2^ | Tarascan | Tarasca |
| 31 | 1943 | J | N | An | F, O | Buena Vista, Ocozotepec, Soteapan Cuilonia, El Tulín, Ocotal Grande, Ocotal Chico, Amamaloya, San Fernando (1); Mirador Palapa, Piedra  Labrada (2); El Aguacate, Horno de Cal, Loma de Sogotegoyo, Loma Larga, Tierra Nueva, Sabaneta, Los Mangos, Barrosa, Soncoavital (3); Comején, Corral  Nuevo (4) | Soteapan (1); Tatahuicapan de  Juárez (2); Hueyapan de Ocampo (3);  Acayucan (4) | Veracruz de Ignacio de la Llave | Sierra Popoluca | Sierra Popoluca | Mixe-Zoque |
| 32 | 1998 | J | Y | Eb | TM, F | Santo Domingo Petapa (1); Santa María Petapa (2); Santa María Guienagati (3); Guevea de Humboldt (4) | Santo Domingo Petapa (1); Santa María Petapa (2); Santa María Guienagati (3); Guevea de  Humboldt (4) | Oaxaca | Isthmus Zapotec | Zapotec | Oto-Mangue |
| 33^4^ | 1983 | J | Y | An | TM | (NS) | Las Margaritas | Chiapas | Tojolabal | Tojolabal | Maya |
| 34 | 2015 | J | Y | Eb | TM | San Miguel Tulancingo | San Miguel Tulancingo | Oaxaca | Chocho | Chocho | Oto-Mangue |
| 35^5^ | 2017 | J | Y | Eb | TM, F, L, O | (NS) | Ocotepec, Tapalapa, Chapultenango, Copainalá, Francisco León, Chiapa de Corzo, Coapilla | Chiapas | Zoque | Zoque | Mixe-Zoque |
| 36^6^ | 2016 | J | Y | Eb | TM | Nuevo Carmen Tonapac (3) | Santa María Chimalapa, San Miguel Chimalapa (1); Ocotepec, Tapalapa, Chapultenango, Copainalá, Francisco León, Coapilla (2), Chiapa de Corzo (3) | Oaxaca (1);  Chiapas (2,3) | Zoque | Zoque | Mixe-Zoque |
| 37 | 2009 | J | N | Eb | TM | (NS) | San José Tenango | Oaxaca | Mazatec | Mazatec | Oto-Mangue |
| 38 | 2005 | J | Y | An | TM | Santo Tomás (1); Chamula (2) | Oxchuc (1); Chamula (2) | Chiapas | Tzotzil (1); Tzeltal (2) | Tzotzil (1);  Tzeltal (2) | Maya |
| 39 | 1998 | J | N | Eb | TM | San Juan Guichicovi | San Juan Guichicovi | Oaxaca | Mixe | Mixe | Mixe-Zoque |
| 40 | 1992 | J | N | Eb | TM | San Juan Guichicovi | San Juan Guichicovi | Oaxaca | Mixe | Mixe | Mixe-Zoque |
| 41 | 1962^3^ | B | N | An | TM, F | Larráinzar (mainly) | Larráinzar | Chiapas | Tzotzil | Tzotzil | Maya |
| 42 | 1970 | J | Y | An | TM, F, Pe, O | Tlayacapan | Tlayacapan | Morelos | Mestizo^2^ | Nahuatl | Yuto-Nahua |
| 43 | 2008 | J | N | An | TM | Atmoloni, Tanamacoyan, Ahuatepec, Nexpan, Dos Ríos, La Aurora (1); Tepetzintán (2) | Hueyapan (1); Cuetzalan del Progreso (2) | Puebla | Northeastern Nahua | Nahuatl | Yuto-Nahua |
| 44 | 1956 | P | Y | An | TM, F | (NS) | (NS) | Oaxaca | Mixtec of the coast of Oaxaca, Mestizo^2^, Afro-Mexican^2^ | Mixtec | Oto-Mangue |
| 45 | 2008 | C | Y | An | TM, O | San Pedro Yosotatu | Heroica Ciudad de Tlaxiaco | Oaxaca | Mestizo^2^ | Mixtec | Oto-Mangue |
| 46 | 1996 | P | Y | Eb | TM, F | San Pedro Yosotatu (mainly) (1); Santa María Yucuhiti (2); Santo Tomás Ocotepec (3); San Pedro Molinos (4); San Andrés Chicahuaxtla (5) | Heroica Ciudad de Tlaxiaco (1); Santa María Yucuiti (2); Santo Tomás Ocotepec (3); San Pedro Molinos (4); Putla Villa de Guerrero (5) | Oaxaca | Mestizo^2^ (1), Mixtec of the central region (east of Putla) (2,3,4);  Trique (5) | Mixteco (1,2,3,4); Trique (5) | Oto-Mangue |
| 47 | 1993 | C | N | Eb | L | San Pedro Yosotatu | Heroica Ciudad de Tlaxiaco | Oaxaca | Mestizo^2^ | Mixtec | Oto-Mangue |
| 48^7^ | 1993 | P | Y | An | TM, F | San Pedro Yosotatu (principalmente) (1); Santa María Yucuhiti (2); Santo Tomás Ocotepec (3); San Pedro Molinos (4); San Andrés Chicahuaxtla (5) | Heroica Ciudad de Tlaxiaco (1); Santa María Yucuiti (2); Santo Tomás Ocotepec (3); San Pedro Molinos (4); Putla Villa de Guerrero (5) | Oaxaca | Mestizo^2^ (1), Mixtec of the central region (east of Putla) (2,3,4);  Trique (5) | Mixtec (1,2,3,4); Trique (5) | Oto-Mangue |
| 49 | 1992 | C | Y | An | TM, F | San Pedro Yosotatu | Heroica Ciudad de Tlaxiaco | Oaxaca | Mestizo^2^ | Mixtec | Oto-Mangue |
| 50^8^ | 1991 | J | Y | An | L, Pe | San Pedro Yosotatu | Heroica Ciudad de Tlaxiaco | Oaxaca | Mestizo^2^ | Mixtec | Oto-Mangue |
| 51 | 1990 | J | Y | Eb | F | San Pedro Yosotatu | Heroica Ciudad de Tlaxiaco | Oaxaca | Mestizo^2^ | Mixtec | Oto-Mangue |
| 52 | 1987 | J | Y | An | TM | (NS) | (NS) | Chihuahua, Sonora | (NS) | (NS) | (NS) |
| 53 | 1971 | B | N | An | F | Santa Catarina Ixtepeji | Santa Catarina Ixtepeji | Oaxaca | Zapotec of the Sierra Juarez | Zapotec | Oto-Mangue |
| 54 | 1976 | B | N | An | TM, F | Tribu Kikapoo (Nacimiento) | Múzquiz | Coahuila | Kickapoo | Kickapoo | Algica |
| 55 | 2015 | C | Y | An | Pe, TM, F | Kopchén (1); Chemax (2) | Felipe Carrillo Puerto (1); Chemax (2) | Quintana  Roo (1); Yucatán (2) | Yucatec Maya | Yucatec | Maya |
| 56 | 2002 | J | Y | Eb | TM | Santa Rosa Loma Larga | Hueyapan de Ocampo | Veracruz de Ignacio de la Llave | Sierra Popoluca | Sierra Popoluca | Mixe-Zoque |
| 57 | 2001 | J | Y | Eb | TM | (NS) | Soteapan, Hueyapan de Ocampo | Veracruz de Ignacio de la Llave | Sierra Popoluca | Sierra Popoluca | Mixe-Zoque |
| 58 | 2020 | J | Y | An | Pe | (NS) | (NS) | Yucatán | Yucatec Maya | Yucatec | Maya |
| 59 | 1960^3^ | B | Y | An | F, TM, L | Tepoztlán | Tepoztlán | Morelos | Center Nahua | Nahuatl | Yuto-Nahua |
| 60 | 1951 | B | Y | An | TM, F, L | Tepoztlán | Tepoztlán | Morelos | Center Nahua | Nahuatl | Yuto-Nahua |
| 61 | 2006 | J | N | An | TM | (NS) | (NS) | Chiapas | Tzotzil (1); Tzeltal (2) | Tzotzil (1);  Tzeltal (2) | Maya |
| 62 | 2012 | J | Y | An | TM, F, Pe, O | Santa Catarina del Monte, San Jerónimo Amanalco | Texcoco | Estado de México | Center Nahua | Nahuatl | Yuto-Nahua |
| 63 | 1955 | J | Y | An | TM, F, Pe, O | San Francisco Tecoxpa | Milpa Alta | Ciudad de México | Center Nahua | Nahuatl | Yuto-Nahua |
| 64 | 1999 | C | Y | Ee | L, TM | Tenejapa (y otras) | Tenejapa | Chiapas | Tzeltal | Tzeltal | Maya |
| 65 | 1959 | J | Y | An | TM, F | San Esteban Atatlahuca (1); 2. San Miguel el Grande (2); Santo Tomás Ocotepec (3) | San Esteban Atatlahuca (1); San Miguel el Grande (2); Santo Tomás Ocotepec (3) | Oaxaca | Mixtec of the central region (east of Putla) | Mixtec | Oto-Mangue |
| 66 | 2008 | C | Y | Eb | TM | Las Flores, Las Nuevas Flores, El Rincón, San Antonio Tancoyol | Jalpan de Serra | Querétaro | Pame | Pame | Oto-Mangue |
| 67 | 1983 | J | Y | An | F, TM | (NS) | (NS) | Oaxaca | (NS) | (NS) | (NS) |
| 68 | 1973 | J | Y | An | TM | (NS) | Ticul | Yucatán | Yucatec Maya | Yucatec | Maya |
| 69 | 1991 | J | Y | Eb | TM | San Pablo Villa de Mitla | San Pablo Villa de Mitla | Oaxaca | Zapotec of the Central Valleys | Zapotec | Oto-Mangue |
| 70 | 1987 | J | Y | An | TM, F | San Pablo Villa de Mitla | San Pablo Villa de Mitla | Oaxaca | Zapotec of the Central Valleys | Zapotec | Oto-Mangue |
| 71 | 1981 | J | Y | An | TM, F | San Pablo Villa de Mitla | San Pablo Villa de Mitla | Oaxaca | Zapotec of the Central Valleys | Zapotec | Oto-Mangue |
| 72 | 1978 | C | Y | Eb | TM, L | San Pablo Villa de Mitla | San Pablo Villa de Mitla | Oaxaca | Zapotec of the Central Valleys | Zapotec | Oto-Mangue |
| 73 | 1975 | J | Y | An | F | Villa Díaz Ordaz | Villa Díaz Ordaz | Oaxaca | Zapotec of the Central Valleys | Zapotec | Oto-Mangue |
| 74 | 2008 | J | N | Eb | L | Santa Catarina Ixtepeji | Santa Catarina Ixtepeji | Oaxaca | Zapotec of the Sierra Juarez | Zapotec | Oto-Mangue |
| 75 | 1967 | J | N | An | TM | Amatenango del Valle | Amatenango del Valle | Chiapas | Tzeltal | Tzeltal | Maya |
| 76 | 1977 | B | Y | An | TM | Santiago Tuxtla (1); San Andrés Tuxtla (2); Catemaco (3) | Santiago Tuxtla (1); San Andrés Tuxtla (2); Catemaco (3) | Veracruz de Ignacio de la Llave | Mestizo^2^ | Nahuatl (1); Sierra Popoluca | Yuto-Nahua (1); Mixe-Zoque (2) |
| 77 | 1985 | J | Y | Eb | TM, F | NE | NE | Oaxaca | Chinantec | Chinantec | Oto-Mangue |
| 78 | 1985 | B | Y | An | F, TM | Motozintla de Mendoza | Motozintla | Chiapas | Mocho | Motocintlec | Maya |
| 79 | 2014 | J | Y | Ee | L | Comachuén | Nahuatzen | Michoacán Michoacán de Ocampo | Purepecha | Tarascan | Tarasca |
| 80 | 2016 | J | N | Eb | TM | La Luna (1), Caxapa (2); Naranjastitla de Victoria (3) | Tezonapa (1,2); San Sebastián  Tlacotepec (3) | Veracruz de Ignacio de la Llave (1,2); Puebla (3) | Mazatec (1,3); Eastern Nahua (2) | Mazatec (1,3); Nahuatl (2) | Oto-Mangue (1,3); Yuto-Nahua (2) |
| 81 | 2017 | B | Y | An | TM | San Mateo Capulhuac | Otzolotipac | Estado de México | Otomi | Otomi | Oto-Mangue |
| 82 | 1941 | B | Y | An | F, TM, Pe, O | Mérida (1); Dzitas (2); Chankom (3); Tuzik (4) | Mérida (1); Dzitas (2); Chankom (3); Felipe Carrillo Puerto (4) | Yucatán (1,2,3); Quintana Roo (4) | Mestizo (1)^2^; Yucatec Maya (2,3,4) | Yucatec (1,2,3,4,) | Maya (1,2,3,4) |
| 83 | 1930 | B | N | An | TM | Tepoztlán | Tepoztlán | Morelos | Center Nahua | Nahuatl | Yuto-Nahua |
| 84 | 1934^3^ | B | Y | An | TM, F, Pe, O | Chankom | Chankom | Yucatán | Yucatec Maya | Yucatec | Maya |
| 85 | 1996 | J | Y | An | F | Xoxocotla | Xoxocotla | Morelos | Center Nahua | Nahuatl | Yuto-Nahua |
| 86 | 1976 | B | Y | An | TM, F, L, O | Huixquilucan de Degollado (mainly) | Huixquilucan | Estado de México | Mestizo^2^ | Otomi | Oto-Mangue |
| 87 | 2012 | B | Y | An | TM | Santa Catarina del Monte | Texcoco | Estado de México | Center Nahua | Nahuatl | Yuto-Nahua |
| 88 | 2008 | J | N | Eb | TM | NS | Nicolás Flores | Hidalgo | Otomi | Otomi | Oto-Mangue |
| 89 | 1982 | J | Y | An | TM, F | Santiago Yancuitlalpan | Cuetzalan del Progreso | Puebla | Northeastern Nahua | Nahuatl | Yuto-Nahua |
| 90 | 1979 | B | Y | An | TM, F | San Mateo del Mar | San Mateo del Mar | Oaxaca | Huave | Huave | Huave |
| 91 | 1963 | C | N | An | TM | NE | Zinacantán | Chiapas | Tzotzil | Tzotzil | Maya |
| 92 | 2012 | J | Y | Eb | TM | Ejido Amatlán^9^ | Ixhuatlán de Madero | Veracruz de Ignacio de la Llave | Huasteca Nahua | Nahuatl | Yuto-Nahua |
| 93 | 2008 | J | Y | Eb | TM | Ejido Amatlán^9^ | Ixhuatlán de Madero | Veracruz de Ignacio de la Llave | Huasteca Nahua | Nahuatl | Yuto-Nahua |
| 94 | 2014 | J | N | Eb | L | Teopisca | Teopisca | Chiapas | Mestizo^2^ | Tzeltal (1); Tzotzil (2) | Maya |
| 95 | 2008 | C | Y | An | TM | (NS) | Xilitla, Axtla de Terrazas, Matlapa, Coxcatlán, Huehuetlán | San Luis Potosí | Huasteca Nahua | Nahuatl | Yuto-Nahua |
| 96 | 2019 | J | Y | Eb | F, L | Las Manzanas | Tenejapa | Chiapas | Tzeltal | Tzeltal | Maya |
| 97 | 2011 | J | Y | Eb | TM | Ahuatla, Xocotla | Coyomeapan | Puebla | Eastern Nahua | Nahuatl | Yuto-Nahua |
| 98 | 1981 | J | Y | An | F, Pe | Chankom (1); Tuzik (2) | Chankom (1); Felipe Carrillo Puerto (2) | Yucatán (1); Quintana  Roo (2) | Yucatec Maya | Yucatec | Maya |
| 99 | 1998 | J | Y | Eb | TM, F, L | (NS) | (NS) | Veracruz de Ignacio de la Llave | Eastern Nahua | Nahuatl | Yuto-Nahua |
| 100 | 1981 | B | Y | An | TM, F, O | San Francisco Pichátaro | Tingambato | Michoacán Michoacán de Ocampo | Purepecha | Tarascan | Tarasca |
| 101 | 1978 | J | Y | An | TM | San Francisco Pichátaro | Tingambato | Michoacán Michoacán de Ocampo | Purepecha | Tarascan | Tarasca |

^1^ The publication does not specify the ethnicity of the human group studied; however, we consider them mestizos because they live in the urbanized areas of Puebla-Tlaxcala.

^2^ Although the main language of the mestizo groups is Spanish, the following cells show the linguistic group(s) and its respective family of origin or that currently influences the mestizo culture.

^3^ Work originally published in this year; does not coincide with the year of the respective reference in Section A.

^4^ Does not specify the study area, but it is possible to make an educated guess at least of the municipality.

^5^ Since the publication did not specify the five municipalities in the study area, the seven municipalities of Chiapas referred to in a previous publication by the same authors (number 35 in this table) were considered.

^6^ The publication indicates that it is a municipality, however, it is a locality.

^7^ The publication mentions that the work was carried out mainly in San Pedro Yosotatu and other unspecified villages, but we assumed that these were the same ones referred to by the author in a later publication (number 46 in this table).

^8^ Although the author did not specify the study area, we assume that it was at least the locality indicated in this table, since it appears in all her subsequent publications (45-49 in this table).

^9^ Unlocated; the municipal seat was located instead.

# Section C. Information on the printed monographs

Publication number (# pub) in correspondence with the list in section A. (NS) = Not specified. The details of the classification and nomenclature of the ethnic groups, languages and language families can be found in the sections G and H. The numbers in parentheses specify corresponding localities, municipalities, states, ethnic groups, linguistic groups and language families.

| **# pub** | **Title** | **Locality(ies)** | **Municipality(ies)** | **State(s)** | **Ethnic group(s) or subgroup(s)** | **Linguistic group(s)** | **Linguistic family(ies)** |
| --- | --- | --- | --- | --- | --- | --- | --- |
| 102 | Popolocas | San Marcos Tlacoyalco(1); San Luis Temalacayuca (2); San Juan Atzingo (3); Los Reyes Metzontla (4); San Felipe Otlaltepec, Almolonga (Todos Santos), Huejonapan (5); Santa Inés Ahuatempan (6); Nativitas Cuautempan, San Vicente Coyotepec (7) | Tlacotepec de Benito Juárez (1); Tepanco de López (2); San Gabriel Chilac (3); Zapotitlán (4) Tepexi de Rodríguez (5); Santa Inés Ahuatempan (6); Coyotepec (7) | Puebla | Popoloca | Popoloca | Oto-Mangue |
| 103 | Mames de Chiapas | (NS) | Acacoyagua, Acapetahua, Amatenango de la Frontera, Bejucal de Ocampo, Bella Vista, Cacahoatán, Escuintla, Frontera Comalapa, Frontera Hidalgo, La Grandeza, Huehuetán, Mazapa de Madero, Mazatán, Metapa, Motozintla, El Porvenir, Villa Comaltitlán, Siltepec, Suchiate, Tapachula, Tuxtla Chico, Tuzantán, Unión Juárez, Maravilla Tenejapa, Las Margaritas and others | Chiapas | Mam | Mam | Maya |
| 104 | Mixes | About 290 (NS) | Santa María Tlahuitoltepec, San Pedro y San Pablo Ayutla, Asunción Cacalotepec, Santa María Tepantlali, Santo Domingo Tepuxtepec, Totontepec Villa de Morelos, Tamazulapam del Espíritu Santo, Mixistlán de la Reforma, San Pedro Ocotepec, Santiago Atitlán, Santa María Alotepec, San Juan Juquila Mixes, San Lucas Camotlán, Santiago Zacatepec, San Miguel Quetzaltepec y Santiago Ixcuintepec, San Juan Mazatlán, San Juan Cotzocón, San Juan Guichicovi | Oaxaca | Mixe | Mixe | Mixe-Zoque |
| 105 | Nahuas de la Huasteca | (NS) | More than 50 (NS) | San Luis Potosí, Hidalgo, Veracruz de Ignacio de la Llave | Huasteca Nahua | Nahuatl | Yuto-Nahua |

# Section D. Information on the sections of the web site “Atlas de los Pueblos Indígenas de México” (http://atlas.inpi.gob.mx)

(NS) = Not specified. The details of the classification and nomenclature of the ethnic groups, languages and language families can be found in the sections G and H. The numbers in parentheses specify corresponding localities, municipalities, states, ethnic groups, linguistic groups and language families.

| **Section** | **Locality(ies)** | **Municipality(ies)** | **State(s)** | **Ethnic group(s) or subgroup(s)** | **Linguistic group(s)** | **Linguistic family(ies)** |
| --- | --- | --- | --- | --- | --- | --- |
| Chichimecas | Mision de Chichimecas | San Luis de la Paz | Guanajuato | Chichimeca | Chichimec | Oto-Mangue |
| Chujes | (NS) | La Trinitaria, La Independencia y Las Margaritas | Chiapas | Chuje | Chuj | Maya |
| Chontales de Oaxaca | (NS) | San Carlos Yautepec, Santa María Ecatepec, Asunción Tlacolulita, San Miguel Ecatepec, Santa Magdalena Tequisistlán, Santiago Astata y San Pedro Huamelula. | Oaxaca | Chontal of Oaxaca | Chontal of Oaxaca | Chontal of Oaxaca |
| Huaves | San Mateo del Mar, Colonia Juárez, Colonia Cuauhtémoc (1); San Francisco del Mar (2); San Dionisio del Mar (3) | San Mateo del Mar (1); San Dionisio del Mar (2); San Francisco del Mar (3) | Oaxaca | Huave | Huave | Huave |
| Mames | (NS) | Acacoyagua, Acapetahua, Amatenango de la Frontera, Bejucal de Ocampo, Bella Vista, Cacahoatán, Escuintla, Frontera Comalapa, Frontera Hidalgo, La Grandeza, Huehuetán, Mazapa de Madero, Mazatán, Metapa, Motozintla, El Porvenir, Villa Comaltitlán, Siltepec, Suchiate, Tapachula, Tuxtla Chico, Tuzantán, Unión Juárez, Maravilla Tenejapa, Las Margaritas (1); Campeche, Champotón (2); Bacalar (3) | Chiapas (1); Campeche (2); Quintana Roo (3) | Mam | Mam | Maya |
| Nahuas de Morelos | 35 localities, the most important are Hueyapan (1), Tetelcingo (2), Santa Catarina (3), Cuentepec (4) y Xoxocotla (5) | Hueyapan(1), Cuautla(2), Tepoztlán (3), Temixco(4), Xoxocotla (5) and other 13 municipalities. | Morelos | Center Nahua | Nahuatl | Yuto-Nahua |
| Nahuas de Hidalgo | (NS) | Principalmente los siguientes: Huejutla de Reyes, San Felipe Orizatlán, Huautla, Yahualica, Atlapexco, Xochiatipan de Castillo, Tlanchinol, Tepehuacán de Guerrero, Tianguistengo, Zimapán (1); Acaxochitlán (2) | Hidalgo | Huasteca Nahua (1); Northeastern Nahua (2) | Nahuatl | Yuto-Nahua |
| Totonacos | (NS) | Ahuacatlán, Amixtlán, Camocuautla, Caxhuacán, Coatepec, Hermenegildo Galeana, Huehuetla, Huauchinango, Hueytlalpan, Atlequizayan, lxtepec, Jalpan, Jopala, Jonotla, Olintla, Pantepec, San Felipe Tepatlán, Tepango de Rodríguez, Tepetzintla, Tlacuilotepec, Tuzamapan de Galeana, Zacatlán, Zapotitlán de Méndez, Zihuateutla, Zongozotla, Zoquiapan (1); Cazones de Herrera, Coahuitlán, Coatzintla, Coxquihui, Coyutla, Chumatlán, Espinal, Filomeno Mata, Mecatlán, Gutiérrez Zamora, Papantla, Tecolutla, Tihuatlán, Zozocolco de Hidalgo (2) | Puebla (1); Veracruz de Ignacio de la LLave(2) | Totonac | Totonac | Totonaco-  Tepehua |
| Popolocas | (NS) | Tlacotepec de Benito Juárez, Tepanco de López, San Gabriel Chilac, Zapotitlán, Tepexi de Rodríguez, Ixcaquixtla, Santa Inés Ahuatempan, Coyotepec | Puebla | Popoloca | popoloca | Oto-Mangue |

# Section E. Information on the sections of the web page "Flora Medicinal Indígena de México" (http://www.medicinatradicionalmexicana.unam.mx/fmim/index.html)

(NS) = Not specified. Superscripts indicate comments at the table footnotes. The details of the classification and nomenclature of the ethnic groups, languages and language families can be found in the sections G and H. The numbers in parentheses specify corresponding localities, municipalities, states, ethnic groups, linguistic groups and language families.

| **Subsection** | **Locality(ies)** | **Municipality(ies)** | **State(s)** | **Ethnic group(s) or subgroup(s)** | **Linguistic group(s)** | **Linguistic family(ies)** |
| --- | --- | --- | --- | --- | --- | --- |
| Flora Medicinal Popoluca de Santa Rosa Loma Larga, Municipio de Hueyapan de Ocampo, Veracruz | Santa Rosa Loma Larga | Hueyapan de Ocampo | Veracruz de Ignacio de la Llave | Sierra Popoluca | Sierra Popoluca | Mixe-Zoque |
| Flora Medicinal Purépecha de Santiago Sicuicho, Municipio Los Reyes de Salgado Michoacán | Sicuicho | Los Reyes | Michoacán de Ocampo | Purepecha | Purepecha | Tarasca |
| Flora Medicinal Rarámuri de Sojahuachi, Chihuahua | Sojahuachi | Bocoyna | Chihuahua | Tarahumara | Tarahumara | Yuto-Nahua |
| Flora Medicinal Seri de Sonora | (NS) | (NS) | Sonora | Seri | Seri | Seri |
| Flora Medicinal Tének de la Comunidad De Tanleab Municipio de Huehuetlan, San Luis Potosí | Tanleab Uno | Huehuetlán | San Luis Potosí | Huastec | Huastec | Maya |
| Flora Medicinal Tepehua de Coyol, Xalame y Chintipan, Municipio de Tlachichilco, Veracruz | Chintipan, El Coyol, Xalame | Tlachichilco | Veracruz de Ignacio de la Llave | Tepehua | Tepehua | Totonaco-  Tepehua |
| Flora Medicinal Tepehuana del Sur de Durango | La Candelaria, La Guajolota, Los Charcos, Santa María de Ocotán (1); San Bernardino de Milpillas, San Francisco de Lajas (Lajas), San Francisco de Ocotán, Santa María Magdalena de Taxicaringa (2) | Mezquital (1); Pueblo Nuevo (2) | Durango | Southern Tepehuan | Southern Tepehuan | Yuto-Nahua |
| Flora Medicinal Totonaca de Papantla de Olarte, Veracruz | Papantla de Olarte | Papantla | Veracruz de Ignacio de la Llave | Totonac | Totonac | Totonaco-  Tepehua |
| Flora Medicinal Triqui de la Laguna Guadalupe, Putla, Oax. | La Laguna Guadalupe | Putla Villa de Guerrero | Oaxaca | Trique | Trique | Oto-Mangue |
| Flora Medicinal Yaqui del Valle del Yaqui, Sonora | (NS) | (NS) | Sonora | Yaqui | Yaqui | Yuto-Nahua |
| Flora Medicinal Zapoteca de Santiago Jalahui, Oaxaca | Santiago Jalahui | San Juan Lalana | Oaxaca | Zapotec of the Sierra Juarez | Zapotec | Oto-Mangue |
| Flora Medicinal Zoque-Popoluca de Magallanes, Veracruz | Magallanes | Tatahuicapan de Juárez | Veracruz de Ignacio de la Llave | Sierra Popoluca | Sierra Popoluca | Mixe-Zoque |
| Flora Medicinal Cora de Jesús María, EL Nayar, Nayarit | Jesús María | Del Nayar | Nayarit | Cora | Cora | Yuto-Nahua |
| Flora Medicinal Guarijía de Sonora^1^ | (NS) | Álamos (1); Uruachi (2) | Sonora (1); Chihuahua (2) | Guarijio | Huarijio | Yuto-Nahua |
| Flora Medicinal Kikapú de Muzquiz, Coahuila | Tribu Kikapoo (Nacimiento) | Múzquiz | Coahuila | Kickapoo | Kickapoo | Algica |
| Floras Medicinales Indígenas de Baja California. Flora Kumiai de San José de la Zorra, Ensenada. | San José de la Zorra | Playas de Rosarito | Baja California | Kiliwa | Kiliwa | Cochimi-  Yumana |
| Floras Medicinales Indígenas de Baja California. Flora Kiliwa de Arrollo de León, Ensenada. | Arroyo de León (Ejido Kiliwas) | Ensenada | Baja California | Kiliwa | Kiliwa | Cochimi-  Yumana |
| Flora Medicinal Mam de Motozintla, Chiapas | Motozintla de Mendoza | Motozintla | Chiapas | Mam | Mam | Maya |
| Flora Medicinal Maya del Estado de Quintana Roo | Santa Rosa Segundo | Felipe Carrillo Puerto | Quintana Roo | Yucatec Maya | Yucatec | Maya |
| Flora Medicinal Mayo de la Región de Ahome, Sinaloa | (NS) | Ahome | Sinaloa | Mayo | Mayo | Yuto-Nahua |
| Flora Medicinal Mayo de la Región de El Fuerte y Choix, Sinaloa | (NS) | El Fuerte, Choix | Sinaloa | Mayo | Mayo | Yuto-Nahua |
| Flora Medicinal Mazahua del Municipio de San Felipe del Progreso, Estado de México | (NS) | San Felipe del Progreso | Estado de México | Mazahua | Mazahua | Oto-Mangue |
| Flora Medicinal Mixteca de Chinango, Oaxaca | Santa Catalina Chinango | San Pedro y San Pablo Tequixtepec | Oaxaca | Puebla Mixtec | Mixtec | Oto-Mangue |
| Flora Medicinal de la Mixteca Alta, Tlaxiaco, Oaxaca | Heroica Ciudad de Tlaxiaco | Heroica Ciudad de Tlaxiaco | Oaxaca | Mixtec of the central region (east of Putla) | Mixtec | Oto-Mangue |
| Flora Medicinal Mixteca de San Pedro Tidaa, Nochistlan, Oaxaca | San Pedro Tidaá | San Pedro Tidaá | Oaxaca | Mixtec of the central region (southeast of Tlaxiaco) | Mixtec | Oto-Mangue |
| Flora Medicinal Nahua de la Magdalena Tlatelulco, Tlaxcala | La Magdalena Tlaltelulco | La Magdalena Tlaltelulco | Tlaxcala | Northeastern Nahua | Nahuatl | Yuto-Nahua |
| Flora Medicinal Nahua de la Sierra de Zongolica, Veracruz | (NS) | (NS) | Veracruz de Ignacio de la Llave | Eastern Nahua | Nahuatl | Yuto-Nahua |
| Flora Medicinal Nahua de San Miguel Tzinacapan, Cuetzalan, Puebla | Tzinacapan | Cuetzalan del Progreso | Puebla | Northeastern Nahua | Nahuatl | Yuto-Nahua |
| Flora Medicinal Otomí de San Juan Ixtenco, Tlaxcala | Ixtenco | Ixtenco | Tlaxcala | Otomi | Otomi | Oto-Mangue |
| Flora Medicinal Otomí de Texcatepec, Veracruz | (NS) | Texcatepec | Veracruz de Ignacio de la Llave | Otomi | Otomi | Oto-Mangue |
| Flora Medicinal Otomí del Valle del Mezquital, Hidalgo | El Bondho, El Sauz, San Antonio Sabanillas, San Miguel Tlazintla, Santa Teresa Daboxtha (1); Orizabita, Dexthí^2^, Cantamaye (2); Taxhay (3) | Cardonal (1); Ixmiquilpan (2); Nicolás Flores (3) | Hidalgo | Otomi | Otomi | Oto-Mangue |
| Floras Medicinales Indígenas de Baja California. Flora Pai-Pai de Santa Catarina, Ensenada | Comunidad Indígena de Santa Catarina | Ensenada | Baja California | Paipai | Paipai | Cochimi-  Yumana |
| Flora Medicinal Pima de Yécora, Sonora | (NS) | Yécora | Sonora | Pima | Pima | Yuto-Nahua |
| Flora Medicinal Popoloca de San Marcos Tlacoyalco y San Juan Atzingo, Puebla | San Juan Atzingo (1); San Marcos Tlacoyalco (2) | San Gabriel  Chilac (1); Tlacotepec de Benito Juárez | Puebla | Popoloca | Popoloca | Oto-Mangue |

^1^ Since the source does not specify the localities or municipalities in the study area, one municipality in each of the states (Sonora and Chihuahua) where the indigenous people studied are distributed was arbitrarily selected to be added to the maps.

^2^ The exact location could not be determined; it could be El Dexthi San Juanito or Puerto Dexthi.

# Section F. Information on the sections of the web page "La Medicina Tradicional de los Pueblos Indígenas de México" (http://www.medicinatradicionalmexicana.unam.mx/mtpim/index.html)

(NS) = Not specified.

Superscripts indicate comments at the table footnotes. The details of the classification and nomenclature of the ethnic groups, languages and language families can be found in the sections G and H. The numbers in parentheses specify corresponding localities, municipalities, states, ethnic groups, linguistic groups and language families.

| **Subsection** | **Locality(ies)** | **Municipality(ies)** | **State(s)** | **Ethnic group(s) or subgroup(s)** | **Linguistic group(s)** | **Linguistic family(ies)** |
| --- | --- | --- | --- | --- | --- | --- |
| Amuzgos. | (NS) | Tlacoachistlahuaca, Xochistlahuaca | Guerrero | Amuzgo | Amuzgo | Oto-Mangue |
| Chatinos (Kitsé Cha Tnio. Kitse Cha’tña. Kitse Tsa’jnya). | (NS) | San Miguel Panixtlahuaca, Santa Catarina Juquila, Villa Sola de Vega | Oaxaca | Chatino | Chatino | Oto-Mangue |
| Chichimeco-Jonaz (Ézar). | Misión de Chichimecas | San Luis de la Paz | Guanajuato | Chichimeca | Chichimec | Oto-Mangue |
| Chinantecos (Tsa ju jmi’). | NE | San Felipe Usila, San Juan Bautista Valle Nacional, San Lucas Ojitlán, San Pedro Sochiápam, Santa María Jacatepec, Santiago Choápam, Santiago Comaltepec, Santiago Nacaltepec | Oaxaca | Chinantec | Chinantec | Oto-Mangue |
| Chochos, Chochones o Chocholtecas. | San Cristóbal Suchixtlahuaca (1); San Juan Bautista Coixtlahuaca (2); Benito Juárez (3);  La Mexicana (4) | San Cristóbal Suchixtlahuaca (1); San Juan Bautista Coixtlahuaca (2); Santa María Nativitas (3); Santiago Ihuitlán Plumas (4) | Oaxaca | Chocho | Chocho | Oto-Mangue |
| Choles (Winikon ba lojon). | Santo Domingo^1^, Frontera Corozal^2^ (1); Conquita^1^, Nueva Esperanza (2) | Ocosingo (1); Tila (2) |  | Ch’ol | Ch'ol | Maya |
| Chontales de Oaxaca o Tequistlatecos. | (NS) | San Carlos Yautepec, Santiago Astata, Santa María Ecatepec | Oaxaca | Chontal of Oaxaca | Chontal of Oaxaca | Chontal of Oaxaca |
| Chujes. | (NS) | La Trinitaria | Chiapas | Chuj | Chuj | Maya |
| Cochimi (M’ti-pa), Cucapá (Es-Pei), Kiliwa (Ko’lew, Koah, Kual), Kumiai (Ti’pai), Paipai (Akwa’ala). Pueblos Indígenas de Baja California.^3^ | San Antonio Necua (Cañada de los Encinos), La Huerta (1); Comunidad Indígena de Santa Catarina (2); Pozas de Arvizu (La Reserva) (3) | Ensenada (1,2); San Luis Río Colorado (3) | Baja California (1,2); Sonora (3) | Cochimi (1); Ku'ahl (2); Cocopah (3) | Cochimi (1); Ku'ahl (2); Cocopa (3) | Cochimi-  Yumana |
| Coras (Nayeri). | (NS) | Del Nayar, Rosamorada | Nayarit | Cora | Cora | Yuto-Nahua |
| Cuicatecos. | (NS) | Concepción Pápalo, San Andres Teotilálpam San Juan Bautista Cuicatlán, San Juan Tepeuxila, Santa María Pápalo, Santos Reyes Pápalo | Oaxaca | Cuicatec | Cuicatec | Oto-Mangue |
| Guarijíos.^4^ | (NS) | Álamos (1); Uruachi (2) | Sonora (1);  Chihuaha (2) | Guarijio | Huarijio | Yuto-Nahua |
| Huastecos (Teenek). | (NS) | Aquismón, Coxcatlán, Huehuetlán, Tamazunchale, Tampamolón Corona, Tancanhuitz, Tantoyuca (1); El Mante (2); Tantoyuca (3) | San Luis Potosí (1); Tamaulipas (2); Veracruz de Ignacio de la Llave (3) | Huastec | Huastec | Maya |
| Huaves, Huazantecos, Juaves, Mareños o Wabis. | Santa María del  Mar (1); San Francisco del Mar Viejo Pueblo Viejo (2) | Juchitán de Zaragoza (1); San Francsico del Mar (2) | Oaxaca | Huave | Huave | Huave |
| Jacaltecos, Cakchiqueles y Motozintlecos o Mochó. | Motozintla de  Mendoza (1); Santa Rita^1^, Pacayalito, Guadalupe Victoria (2); Nuevo Amatenango, Tapitzalá Número Dos (3); Paso Hondo (4); Agua Zarca (5); La Junta^1^ (6) | Motozintla (1); Amatenango de la Frontera (2,3); Frontera Comalapa (4,5); Mazapa de Madero (6) | Chiapas | Mocho (1); Jakaltek (2, 4) Kaqchikel; (3,5,6) | Motocintlec (1); Jacaltec (2,4); Cakchiquel (3,5,6) | Maya |
| Huicholes (Wirraritari). | El Naranjo (1); Guineas de Guadalupe (2); La Paloma Primera (3); Santa Bárbara, Tepic^5^, Zoquiapan del Nayar (4) | Ruiz (1); Del Nayar (2);  Acaponeta (3); Tepic (4) | Nayarit | Huichol | Huichol | Yuto-Nahua |
| Ixcatecos. | Santa María Ixcatlán | Santa María Ixcatlán | Oaxaca | Ixcatec | Ixcatec | Oto-Mangue |
| Mames. | (NS) | El Porvenir, Siltepec | Chiapas | Mam | Mam | Maya |
| Matlatzincas y Ocuiltecos: Tlahuicas.^6^ | Santa María Nativitas, San Juan Atzingo, Santa Mónica | Ocuilán | Estado de México | Ocuiltec | Ocuiltec | Oto-Mangue |
| Mayas. | (NS) | Chacsinkín, Chankom, Chemax, Chichimilá, Chikindzonot, Halachó, Peto, Sotuta, Tahdziú, Tekom, Tixcacalcupul, Tixméhuac, Tzucacab, Valladolid, Yaxcabá (1); Calkiní (2); Hopelchén (3) | Yucatán (1); Campeche (2); Quintana Roo (3) | Yucatec Maya | Yucatec | Maya |
| Mayos (Yoreme). | (NS) | Etchojoa, Huatabampo, Navojoa (1); Ahome, Choix, El Fuerte, Guasave (2) | Sonora (1);  Sinaloa (2) | Mayo | Mayo | Yuto-Nahua |
| Mazahuas. | (NS) | Atlacomulco, El Oro, Temascalcingo, San Felipe del Progreso, Villa de Allende (1); Zitácuaro (2) | Estado de México (1);  Michoacán de Ocampo (2) | Mazahua | Mazahua | Oto-Mangue |
| Mazatecos. | (NS) | Acatlán de Pérez Figueroa, Chiquihuitlán de Benito Juárez, Huautla de Jiménez, San José Independencia, San Miguel Soyaltepec, San Pedro Ixcatlán, Santa María Chilchotla (1); San Sebastián Tlacotepec (2) | Oaxaca (1); Puebla (2) | Mazatec | Mazatec | Oto-Mangue |
| Mexicaneros y Tepehuanes del Sur (O’dam). | (NS) | Huajicori, Acaponeta (1); Guadalupe y Calvo (2) | Nayarit (1); Chihuahua (2) | Western Nahua (1); Southern Tepehuan(1); Northern Tepehuan (2) | Nahuatl (1); Southern Tepehuan (1); Northern Tepehuan (2) | Yuto-Nahua |
| Mixes (Ayuuk Jä’Äy). | (NS) | Asunción Cacalotepec, Mixistlán de la Reforma, San Juan Guichicovi, San Miguel Quetzaltepec, Santa María Alotepec, Santa María Tlahuitoltepec | Oaxaca | Mixe | Mixe | Mixe-Zoque |
| Mixtecos. | (NS) | Matías Romero^7^ (1); Pinotepa de Don Luis, San Agustín Chayuco, Santa María Huazolotitlán, Santiago Jamiltepec (2); Cuyamecalco Villa de Zaragoza, San Miguel Huautla, San Pedro Jaltepetongo, San Pedro Jocotipac, Santa María la Asunción, Santa María Texcatitlán, Santiago Nacaltepec (3); San Juan Mixtepec, San Miguel Tlacotepec, Silacayoápam (4); San Esteban Atatlahuca, San Pedro Molinos, Santiago Nuyoó (5); San Martín Huamelúlpam, San Pedro Tidaá, Santa Catarina Tayata (6); Atlamajalcingo del Monte (7); Metlatónoc (8); Concepción Buenavista (9); Caltepec (10) | Oaxaca (1,2,3,4,5,6,9); Guerrero (7,8);  Puebla (10) | Mixtec of the coast of Oaxaca (2); Northeastern Mixtec (3); Western Mixtec (4); Mixtec of the central region (east of Putla) (5); Mixtec of the central region (southeast of Tlaxiaco) (6); Mixtec of northern Guerrero (7); Mixtec of southern Guerrero (8); Puebla Mixtec (9,10) | Mixtec | Oto-Mangue |
| Nahuas. | (NS) | Atlapexco, Huautla, Huazalingo, Huejutla de Reyes, Xochiatipan de Castillo, Yahualica (1); Atempan, Chichiquila, Chiconcuautla, Chilchotla, Cuautempan, Cuetzalan del Progreso, Huauchinango, Hueytamalco, Ixtacamaxtitlán, Ixtamaxtitlán^1^, Lafragua, Naupan, Quimixtlán, Teziutlán, Tlaola, Tlatlauquitepec, Tuzamapan de Galeana, Zacapoaxtla (2); Tlacoxitlahuaca^1^, Chilapa de Álvarez, Olinalá (3); Acaponeta, Huajicori (4); Aquila, Coahuayana (5); Texhuacán, Zongolica (6) | Hidalgo (1);  Puebla (2);  Guerrero (3);  Nayarit (4); Michoacán de Ocampo (5); Veracruz de Ignacio de la Llave (6) | Huasteca Nahua (1); Northeastern Nahua (2); Center Nahua (3); Western Nahua (4,5); Eastern Nahua (6) | Nahuatl | Yuto-Nahua |
| Otomies (Nyühü’ o Hña’ñü). | (NS) | Acambay de Ruiz Castañeda (1); Huehuetla, Ixmiquilpan, Nicolás Flores, Tenango de Doria (2); Zitácuaro (3); Pahuatlán (4); Amealco de Bonfil, Cadereyta de Montes, Tolimán (5) | Estado de México (1); Hidalgo (2); Michoacán de Ocampo (3);  Puebla (4);  Querétaro (5) | Otomi | Otomi | Oto-Mangue |
| Pames (Xi’oi o Xiyoi). | (NS) | Alaquines, Ciudad del Maíz, Rayón, Santa Catarina, Tamasopo | San Luis Potosí | Pame | Pame | Oto-Mangue |
| Papagos (Tono-Ooh’tam). | (NS) | Pitiquito, General Plutarco Elías Calles | Sonora | Tohono Oʼodham | Tohono Oʼodham | Yuto-Nahua |
| Pima (O’ob). | (NS) | Temosáchic (1); Yécora (2) | Chihuahua (1);  Sonora (2) | Pima | Pima | Yuto-Nahua |
| Popolocas. | (NS) | Tepexi de Rodríguez, Tlacotepec de Benito Juárez | Puebla | Popoloca | Popoloca | Oto-Mangue |
| Purépechas. | (NS) | NE | Michoacán de Ocampo | Purepecha | Purepecha | Purepecha |
| Seri (Konkaak). | Punta Chueca (1); Desemboque de los Seris (El  Desemboque) (2) | Hermosillo (1); Pitiquito (2) | Sonora | Seri | Seri | Seri |
| Tarahumaras (Raramuris). | (NS) | Balleza, Batopilas de Manuel Gómez Marín, Bocoyna, Carichí, Guachochi, Guadalupe y Calvo, Urique | Chihuahua | Tarahumara | Tarahumara | Yuto-Nahua |
| Tepehuas (Hamaispini, Kitndnkanmakalkaman). | Mecapalapa^8^ (1) | Pantepec (1); Ixhuatlán de Madero, Tlachichilco (2) | Puebla (1);  Veracruz de Ignacio de la Llave (2) | Tepehua | Tepehua | Totonaco-  Tepehua |
| Tlapanecos (Mbo Me’phaa). | (NS) | Atlamajalcingo del Monte, Malinaltepec, Metlatónoc | Guerrero | Tlapanec | Tlapanec | Oto-Mangue |
| Tojolabales (Tojolwinik’otik). | (NS) | Las Margaritas | Chiapas | Tojolabal | Tojolabal | Maya |
| Totonacos. | (NS) | Amixtlán, Ayotoxco de Guerrero, Hermenegildo Galeana, Tenampulco, Zapotitlán de Méndez, Zihuateutla (1); Coxquihui, Espinal, Papantla, Tihuatlán (2) | Puebla (1);  Veracruz de Ignacio de la Llave (2) | Totonac | Totonac | Totonaco-  Tepehua |
| Triquis (Tinujei). | (NS) | Putla Villa de Guerrero, San Martín Itunyoso, Santiago Juxtlahuaca | Oaxaca | Trique | Trique | Oto-Mangue |
| Tzeltales (Winik Atel). | (NS) | Berriozábal, Ocosingo, Oxchuc, Teopisca, Venustiano Carranza | Chiapas | Tzeltal | Tzeltal | Maya |
| Tzotziles (Bats’il Winik). | (NS) | Bochil, Chamula, Chenalhó, Chicoasén, Coapilla, Comitán de Domínguez, El Bosque, Larráinzar, Ocozocoautla de Espinosa , Pueblo Nuevo Solistahuacán, Venustiano Carranza | Chiapas | Tzotzil | Tzotzil | Maya |
| Yaquis (Yoreme). | (NS) | Bácum, Cajeme, Guaymas | Sonora | Yaqui | Yaqui | Yuto-Nahua |
| Zapotecos (Bene Xon. Ben’zaa. Binniza). | (NS) | Ixtlán de Juárez, San Juan Juquila Vijanos, Santiago Choápam, Santiago Yaveo, Talea Villa de Castro (1); San Andrés Ixtlahuaca (2); Guevea de Humboldt, San Pedro Huamelula, Santa María Guienagati, Santiago Laollaga, Santo Domingo Petapa (3) | Oaxaca | Zapotec of the Sierra Juarez (1); Zapotec of the Central Valleys (2); Isthmus Zapotec (3) | Zapoteco | Yuto-Nahua |
| Zoques de Chiapas (O’de Put). | (NS) | Chapultenango, Chicoasén, Copainalá, Ocotepec, Ostuacán, Pichucalco, Rayón, Tapilula, Tecpatán (1); San Miguel Chimalapa (2) | Chiapas (1);  Oaxaca (2) | Zoque | Zoque | Mixe-Zoque |
| Zoque-Popoluca. | (NS) | Hueyapan de Ocampo, Mecayapan | Veracruz de Ignacio de la Llave | Sierra Popoluca | Sierra Popoluca | Mixe-Zoque |

^1^ Unlocated.

^2^ The original source states that this locality belongs to the municipality of Sabanilla, but in reality, it belongs to the municipality of Ocosingo.

^3^ The source mentions the localities where the six indigenous peoples covered in the monograph are distributed (the Ku'ahl group was added during fieldwork), but did not specify the localities of the concrete study area, except for the Cocopah group. Since three of them (Cochimi, Cocopah and Ku'ahl) had not been recorded in any other source, we decided to include some of their localities in the maps. Two localities were arbitrarily chosen for the Cochimi; of the Cocopah, the locality studied, and of the Ku'ahles, the only locality mentioned in the monograph.

^4^ Since the source does not specify the localities or municipalities in the study area, one municipality in each of the states (Sonora and Chihuahua) where the indigenous people are distributed was arbitrarily selected for the maps.

^5^ The exact location, according to the original source, was Colonia Zitakua in the City of Tepic.

^6^ According to the *Atlas of Indigenous Peoples of Mexico* (reference 106 in Section A), the Matlatzincas live in the community of San Francisco Oxtotilpan (municipality of Temascaltepec, State of Mexico), which was not covered in the monograph.

^7^ This municipality is completely outside the original distribution area of the Mixtec people, perhaps the informants interviewed were migrants.

^8^ In the original source it is mentioned as a municipality.

# Section G. List of linguistic and ethnic groups of Mexico using the hot-cold system

Nomenclature in English of the linguistic groups and endonyms of linguistic variants according to the *Catalog of National Indigenous Languages.* The symbol † indicates that the linguistic group is extinct. The name of each ethnic group is in English and is only one of the ways in which it has been recorded in the literature. Endonyms of the ethnic groups according to Valiñas Coalla (2020) and the *Atlas of Indigenous Peoples of Mexico*. The source numbers correspond to the list in Section A. For sources that are websites, the number in parenthesis indicates individual sections with information, when there are two or more.

| **Linguistic familiy** | **Linguistic group** | **Endonym(s) of linguistic variants** | **Ethnic group** | **Endonym(s) of the ethnic group** | **Source number(s)** |
| --- | --- | --- | --- | --- | --- |
| I. Algica | Kickapoo | kickapoo | Kickapoo | kikaapoa, kikapua (singular), kikapuaki (plural) | 54, 107 |
| II. Yuto-Nahua | Tohono O’odham | tohono o’otham, tohono o’odham | Tohono Oʼodham | tohono o’otham | 108 |
| II. Yuto-Nahua | Pima | oob no’ok, obnók | Pima | o’ob, oob | 107, 108 |
| II. Yuto-Nahua | Northern Tepehuan | odami | Northern Tepehuan | óódame, ódame | 108 |
| II. Yuto-Nahua | Southern Tepehuan | au'dam, o'dam | Southern Tepehuan | o’dam, au’dam | 107, 108 |
| II. Yuto-Nahua | Tepecano † | (not available) | Tepecano | (not available) |  |
| II. Yuto-Nahua | Tarahumara | rarómari raicha, ralámuli raicha, rarámari raicha | Tarahumara | rarámuri, ralámuli, ralómali, ralamuli, ralomli, ralamali | 107, 108 |
| II. Yuto-Nahua | Huarijio | warihó, makurawe | Guarijio | macurawe, warihó, macoragüi, maculái, macurái | 107, 108 |
| II. Yuto-Nahua | Tubar † | (not available) | Tubar | (not available) |  |
| II. Yuto-Nahua | Yaqui | hiak nooki | Yaqui | yoreme | 108 |
| II. Yuto-Nahua | Mayo | yorem-nokki | Mayo | yoreme | 107(2), 108 |
| II. Yuto-Nahua | Opata † | (not available) | Opata | (not available) |  |
| II. Yuto-Nahua | Eudeve † | (not available) | Eudeve | (not available) |  |
| II. Yuto-Nahua | Cora | rosaríìtu, wachi hapwa, yaúhke’ena, chwísita’na, kwáaxa’ata, kwéimarusa’na, quamaruchi, múxata’ana, kuráàpa | Cora | náayari, nayeri, yaúhke’ena, yohke, chwísita’na, ahusete | 107, 108 |
| II. Yuto-Nahua | Huichol | wixárika | Huichol | wixárika, wirrárika (singular); wixaritari, wirraritari (plural) | 108 |
| II. Yuto-Nahua | Pochutec † | (not available) | Pochutec | (not available) |  |
| II. Yuto-Nahua | Nahuatl | (see Section H) | Nahua | (not available) | 4, 5, 11, 19, 20, 43, 59, 60, 62, 63, 80, 83, 85, 87, 89, 92, 93, 95, 97, 99, 105, 106(2), 107(3), 108(2) |
| III. Cochimi-Yumana | Kumeyaay | tipai | Kumeyaay | kumiay, ti’pai, laymon, diegueños, m’ti-pa, tiipay, kumeyaay | 107, 108 |
| III. Cochimi-Yumana | Cocopa | kuapá | Cocopah | es-pei, kuapá, xawitt kwñchawaay | 108 |
| III. Cochimi-Yumana | Paipai | jaspuy pai | Paipai | (not available) | 107, 108 |
| III. Cochimi-Yumana | Akwa’ala | ku’ahl | Ku'ahl | (not available) | 108 |
| III. Cochimi-Yumana | Kiliwa | ko’lew | Kiliwa | ko’lew, ko lew | 107, 108 |
| III. Cochimi-Yumana | Cochimi † | (not available) | Cochimi | comom’ti-pa | 108 |
| IV. Seri | Seri | cmiique iitom | Seri | könkáak, konkaak, conca’ac, comcáac | 107, 108 |
| V. Oto-Mangue | Pame | xi’iuy, xi’oi | Pame | (not available) | 66, 108 |
| V. Oto-Mangue | Chichimec | uzá’ | Chichimeca | úza’ (singular), éza´r (plural) chichimecos jonaces, chichimecos, mecos | 106, 108 |
| V. Oto-Mangue | Otomi | ñuju, ñoju, yühu, hñäñho, hñöñho, ñühú, ñänhú, ñathó, ñöhñö, ñähñá, hñähñú, ñandú, ñóhnño, ñanhmu, yühmu, nü’hü, hñähñu, ñöthó, ñható, hñothó, ñanhú | Otomi | (not available) | 81, 88, 107(3), 108 |
| V. Oto-Mangue | Mazahua | jnatrjo, jnatjo | Mazahua | jñatrjo, ñatjo, jnatrjo, jnatjo | 107, 108 |
| V. Oto-Mangue | Matlatzinca | bot’una | Matlatzinca | matlatzincas |  |
| V. Oto-Mangue | Ocuiltec | pjiekakjoo | Ocuiltec | (not available) | 108 |
| V. Oto-Mangue | Chinantec | jujmi, jujmi tsa kö’w++, jmiih kia’ dzä ‘vï’ï , j+g ki tsomän, j+g dsa k+, jumi dsa iin+n, jumi dsa mojai, jujmi dsa maji’i, jujmi dsa m+ta’o, jaú jm, jmiih kia’ dzä jii’, jmiih dzä mo’, jau jmai, jujma, jejmei, jejmi, jajmi dzä kï’ï, jujmi, jajme dzä mii, jmiih kia’ dzä mii (cat.) | Chinantec | tsa ju jmí | 15, 77, 108 |
| V. Oto-Mangue | Tlapanec | me'phaa tsíndíí, tlapaneco, me'phaa xkua ixi ridií, me'paa wí'i in, me'phaa bátháá, mi'phaa míŋuíí, me'phaa xirágáá, me'phaa aguaa, me'phaa xma'íín | Tlapanec | (not available) | 108 |
| V. Oto-Mangue | Chiapanec † | (not available) | Chiapanec | (not available) |  |
| V. Oto-Mangue | Mazatec | en naxijen, en ningotsie, ntaxjo, nne nangui ngaxni, enre naxinanda nguifi, en ngixo, enna, ienra naxinandana nnandia, an ndexu, en naxo tota, en ndaja, en naxi llanazo, an xo’boo | Mazatec | ha shuta enima | 37, 80; 108 |
| V. Oto-Mangue | Ixcatec | xwja | Ixcatec | xhwani | 108 |
| V. Oto-Mangue | Chocho | ngiba, ngigua | Chocho | runixa ngiigua, ngiwa, ngigua | 34 |
| V. Oto-Mangue | Popoloca | ngiba, ngiwa, ngigua | Popoloca | ngiwa, ngigua | 1, 102, 106 107, 108 |
| V. Oto-Mangue | Zapotec | (see Section H) | Zapotec | (not available) | 22, 32, 53, 69-73, 107, 108 (2) |
| V. Oto-Mangue | Chatino | cha’ jna’a | Chatino | kitsé cha’tnio, kitse cha’tña, kitse tsa’jnya | 108 |
| V. Oto-Mangue | Amuzgo | nomndaa, ñomndaa, nta, nundá’, ts’unuma, jñunnda, jnòn ndá tsjóon nuàn, jñon’ndaa, ñonndaa, ñonndá | Amuzgo | nn´anncue | 108 |
| V. Oto-Mangue | Mixtec | (see Section H) | Mixtec | (not available) | 10, 16, 17, 46, 48, 65, 107 (3), 108 |
| V. Oto-Mangue | Cuicatec | dbaku, dibaku, duaku, dubaku | Cuicatec | dibaku | 108 |
| V. Oto-Mangue | Trique | xnánj nu’ a, stnáj ni’, gui a’mi nánj nï’ïn, tnanj ni’inj | Trique | (not available) | 46, 48, 107, 108 |
| VI. Maya | Huastec | teenek, tenek | Huastec | teenek, tenek | 3, 107, 108 |
| VI. Maya | Chicomuseltec † | (not available) | Chicomuseltec | (not available) |  |
| VI. Maya | Yucatec | maaya, maaya t’aan, maayáa | Yucatec Maya | mayas | 8, 9, 13, 55, 58, 68, 82, 84, 98, 107, 108 |
| VI. Maya | Lacandon | jach-t’aan | Lacandon | hach winik, jach-t’aan, lakantún |  |
| VI. Maya | Chontal | yoko t’an | Chontal Maya | yoko t’anob, yoko t’an, yokot'an |  |
| VI. Maya | Ch'ol | lakty’añ | Ch’ol | winik, xixik | 108 |
| VI. Maya | Tzeltal | bats’il k’op | Tzeltal | winik atel | 38, 61, 64, 75, 96, 108 |
| VI. Maya | Tzotzil | bats’i k’op | Tzotzil | bats’il winik | 38, 41, 61, 91, 108 |
| VI. Maya | Kanjobal | k’anjob’al | Kanjobal | q’anjob’al, q’aanjob’al |  |
| VI. Maya | Acatec | kuti’ | Acatec | kuti’ |  |
| VI. Maya | Jacaltec | jakalteko-popti’ | Jakaltek | jakalteko-popti | 107, 108 |
| VI. Maya | Motocintlec | mocho’, muchu’ | Mocho | mochós, motozintlecos | 78, 107, 108 |
| VI. Maya | Tojolabal | tojol-ab’al | Tojolabal | tojolab’al, tojol-ab’al | 12, 33, 108 |
| VI. Maya | Chuj | koti’ | Chuj | chonhab’ kob’a | 106, 108 |
| VI. Maya | Quiché | k’iche’ | Quiché | k’iche’ |  |
| VI. Maya | Cakchiquel | kaqchikel | Kaqchikel | kaqchikel | 107, 108 |
| VI. Maya | Kekchí | q’eqchi’ | Kekchi | q’eqchi’ |  |
| VI. Maya | Tectitec | qyool, b’a’aj | Teko | qyool, b’a’aj |  |
| VI. Maya | Mam | qyool, qyool mam, b’anax mam | Mame | winaq qo’ | 103, 106, 107, 108 |
| VI. Maya | Aguacatec | qyool | Awakatek | qatanum |  |
| VI. Maya | Ixil | ixil | Ixil | (not available) |  |
| VII. Totonaco-Tepehua | Totonac | laakanaachiwíin, tachaqawaxti, tutunakuj, tachiwiin, kintachiuinkan, tutunáku, lichiwin tutunaku, totonaco, tutunakú | Totonac | (not available) | 106, 107, 108 |
| VII. Totonaco-Tepehua | Tepehua | lhiimaqalhqama’, lhiima’alh’ama, lhichiwíin, lhiimaasipijni o liimaasipijni | Tepehua | (not available) | 107, 108 |
| VIII. Tarasca | Tarascan | p’urhepecha, p’orepecha | Purepecha | p’urhépecha, p’urhepecha, p’orepecha | 79, 100, 101, 107, 108 |
| IX. Mixe-Zoque | Mixe | ayöök, ayuujk, ayuuk, eyuk, ayuk | Mixe | ayöök | 39, 40, 104, 108 |
| IX. Mixe-Zoque | Tapachultec † | (not available) | Tapachultec | (not available) |  |
| IX. Mixe-Zoque | Sayultec | t+kmaya’, yámay | Sayula Popoluca | (not available) |  |
| IX. Mixe-Zoque | Olutec | yaakaw+ | Oluta Popoluca | (not available) |  |
| IX. Mixe-Zoque | Zoque | tsuni, ode, ore, ote, angpø’n, angpø’n tsaame | Zoque | o’ de püt | 35, 36, 108 |
| IX. Mixe-Zoque | Sierra Popoluca | nuntaj±yi’, nunta anh+maatyi | Sierra Popoluca | (not available) | 20, 31, 56, 57, 107, 108 |
| IX. Mixe-Zoque | Ayapanec | numte oote | Ayapanec | numte oote | 6 |
| IX. Mixe-Zoque | Texistepec Zoque | wää 'oot | Texistepec Popoluca | (not available) |  |
| X. Chontal of Oaxaca | Chontal of Oaxaca^1^ | tsame, tsome, lajltyaygi | Chontal of Oaxaca | slijuala xanuc’, lopimaye | 106, 108 |
| XI. Huave | Huave | ombeayiüts, ombeyajts | Huave | ikoots, ikojts, kunajts | 90, 106, 108 |

^1^We have chose this name because the official catalog lists only the linguistic variants (Huamelultec, Lowland Chontal, Tequistlatec, Highland Chontal).

# Section H. List of Nahua, Zapotec and Mixtec ethnic groups using the hot-cold system

Endonyms of linguistic variants according to the *Catalog of National Indigenous Languages*. The source numbers correspond to the list in Section A. For sources that are websites, the number in parenthesis indicates individual sections with information, when there are two or more.

| **Linguistic familiy** | **Linguistic group** | **Endonym(s) of linguistic variants** | **Ethnic group** | **Source number(s)** |
| --- | --- | --- | --- | --- |
| II. Yuto-Nahua | Nahuatl | mexcatl, náhuatl, mexicano, mexicatl | Huasteca Nahua | 92, 93, 95, 105, 106, 108(2) |
| II. Yuto-Nahua | Nahuatl | mexicano tlajtol, nauta, mexi’catl, mexicano, masehual tla’tol, maseual tajtol, nahuat | Northeastern Nahua | 11, 43, 89, 106, 107(2), 108 |
| II. Yuto-Nahua | Nahuatl | mexicano, náhuatl, mexicatl | Eastern Nahua | 80, 97, 99, 108 |
| II. Yuto-Nahua | Nahuatl | náhuatl, mexicano | Southern Nahua | 20 |
| II. Yuto-Nahua | Nahuatl | mexicano | Center Nahua | 4, 5, 19, 59, 60, 62, 63, 83, 85, 87, 106, 108, 107 |
| II. Yuto-Nahua | Nhuatl | mexicano | Western Nahua | 108(2) |
| V. Oto-Mangue | Zapotec | diste’, distèe, desté, ditsa’, ristee’, didsé, ditsa, ditsè, didxke’, dixhque', didxnde, dichsà, ditsáh | Coastal Zapotec |  |
| V. Oto-Mangue | Zapotec | dixazà, diidxazá, diixazá, didxsá, ditsá, ditsire, di’tsiri’e, didxazé, dizazi, ditsere’, dixhtió | Isthmus Zapotec | 32, 108 |
| V. Oto-Mangue | Zapotec | rixhquei, rizi, risna, rixhna, di’tse’e, dialó, diiste, dizde, dizè, dizhze, distse’, ditsè, ditsëë, ditsë, didxnde, diste', distse'e, disà, di'izhdë, tixh nguizë | Zapotec of the Sierra Sur |  |
| V. Oto-Mangue | Zapotec | tizha', tizhà, tizáriù, xhtisariú, didzá, tidza', xitzhá, diza shisa', diza shitsa, diza shitsá, di' shitsá, xitsúa, didxa shiza', didza shon, dilla xidza, dishuràsh, diya xhon, didza shon, dilla xhon, dille xhon, diya dxon, dilla rhune', ditse', tizha | Zapotec of the Sierra Juarez | 22, 53, 107, 108 |
| V. Oto-Mangue | Zapotec | dichsah, didxazá, dixdà, diza, disàa, dzhi’iza, dixzhá, disá, dizdà, dizhá, dizá, dixasà, didzé, dizë, tizá, dixsà, dizé, dizè, xtizhna | Zapotec of the Central Valleys | 69-73, 108 |
| V. Oto-Mangue | Mixtec | tno’on sàvi, jnu’un sábi, tno’on sawi, tu’un savi | Mixtec of northern Guerrero | 17, 108 |
| V. Oto-Mangue | Mixtec | tu’un Isasi, tu’un savi, tno’on savi, tu'un isavi | Mixtec of southern Guerrero | 108 |
| V. Oto-Mangue | Mixtec | tu’un savi, to’on savi, tu’un va’a | Mixtec of the coast of Oaxaca | 108; 44 |
| V. Oto-Mangue | Mixtec | tu’un savi, tu’un davi, tu’un ñudavi, tu’un lavi, tu’un dau, tnu’un davi | Eastern Mixtec | 16 |
| V. Oto-Mangue | Mixtec | tu’un davi, tu’un savi, da’an davi | Northeastern Mixtec | 108 |
| V. Oto-Mangue | Mixtec | sa'an savi, tu’un savi, tu’un djavi, tu’un javi, dedavi, sa'an sau | Northwestern Mixtec |  |
| V. Oto-Mangue | Mixtec | da'an davi, de'e dau | Puebla Mixtec | 107, 108 |
| V. Oto-Mangue | Mixtec | sa'an ntavi, to'on savi, tu'un da'vi, to'on nda'vi | Western Mixtec | 108 |
| V. Oto-Mangue | Mixtec | tu’un savi, sa’an sau, sasau, tnu’u ñuu savi, sahin sau | Mixtec of the central region (east of Putla) | 10, 46, 48, 65, 107, 108 |
| V. Oto-Mangue | Mixtec | tu’un savi, dañudavi, tnu’un dawi, tnu’un dau, tnu’u savi | Mixtec of the central region (southeast of Tlaxiaco) | 16, 107, 108 |
